# Women's experiences of maternity care in high-income countries during the pandemic health system shock: a follow-up systematic review and qualitative evidence synthesis

**DOI:** 10.3389/fpubh.2026.1715725

**Published:** 2026-04-22

**Authors:** Camila Carbajal, Tisha Dasgupta, Emily Russell, Su Mon Latt, Gillian Horgan, Lili Peterson, Hiten D. Mistry, Kirsty Kitchen, Milly Wilson, Valerie Smith, Harriet Boulding, Kayleigh S. Sheen, Aricca D. Van Citters, Eugene C. Nelson, Emma L. Duncan, Peter von Dadelszen, The RESILIENT Study Group, Sergio A. Silverio, Laura A. Magee

**Affiliations:** 1 Department of Women & Children's Health, School of Life Course & Population Sciences, King's College London, London, United Kingdom; 2 Department of Population Health Sciences, School of Life Course & Population Sciences, King's College London, London, United Kingdom; 3 The RESILIENT Study Patient & Public Involvement & Engagement Advisory Group, United Kingdom; 4 Birth Companions, London, United Kingdom; 5 School of Nursing, Midwifery & Health Systems, College of Health and Agricultural Sciences, University College Dublin, Dublin, Ireland; 6 The Policy Institute, Faculty of Social Science & Public Policy, King's College London, London, United Kingdom; 7 Department of Social Sciences, College of Health, Science and Society, University of the West of England Bristol, Bristol, United Kingdom; 8 The RESILIENT Study Technical Advisory Group, United Kingdom; 9 The Dartmouth Institute for Health Policy & Clinical Practice, Geisel School of Medicine, Dartmouth College, Hanover, NH, United States; 10 Department of Twin Research & Genetic Epidemiology, School of Life Course & Population Sciences, King's College London, London, United Kingdom; 11 The RESILIENT Study Group, United Kingdom; 12 Department of Psychology, Institute of Population Health, University of Liverpool, Liverpool, United Kingdom

**Keywords:** COVID-19, high-income countries, maternity care, pregnancy, qualitative research, systematic review, women

## Abstract

**Introduction:**

COVID-19 disrupted healthcare systems globally, particularly challenging maternity services which continued to be operated as an essential service. Reconfigurations were implemented to continue providing care in a safe manner and in line with infection control restrictions. This systematic review of women's experiences of maternity care during the COVID-19 pandemic in high-income countries (HICs), aimed to synthesize published literature and inform future responses to global disasters.

**Material and methods:**

Electronic database of Scopus, MEDLINE, EMBASE, CINAHL PsychINFO, and the Cochrane COVID Study Register, were searched from June 2021- June 2024 to identify eligible records. Thematic synthesis was used to synthesise the data.

**Results:**

79 studies were included with data from over 20,000 perinatal women, most were of moderate to high methodological quality. Data synthesis showed 11 themes across five main concepts related to maternity service reconfigurations, namely: (1) Care-seeking and care experience, (2) Virtual care, (3) Self-monitoring, (4) Vaccination, and (5) Ethical future of maternity care.

**Conclusions:**

Women predominantly viewed changes to maternity care negatively. Future strategies to ensure safeguarding of mothers and infants during crises should include enhancing service accessibility, emphasizing women-centered care, and prioritizing support systems for mothers and infants.

**Systematic review registration:**

https://www.crd.york.ac.uk/PROSPERO/view/CRD42022355948, identifier: CRD42022355948.

## 1 Introduction

The COVID-19 pandemic has had a dramatic and long-lasting impact on healthcare globally, with over 777 million cases (1) and 7.1 million deaths reported, likely to be an underestimation (2). Maternity services continued to be delivered throughout and whilst pregnant women were not at higher risk of contracting COVID-19, they were more prone to develop severe illness, especially in the third trimester (3), leading to increased iatrogenic preterm births, cesarean sections, and Intensive Care Unit (ICU) admissions (4). Enforced lockdowns, social isolation, and loss of support worsened pregnancy challenges (5), hindering care-seeking and exacerbating psychological vulnerability. Social distancing predicted significant risks of maternal depression (30%) and anxiety (33%), after accounting for clinical diagnosis and demographic factors (6).

Globally, countries implemented protective measures, encouraging self-isolation, and widespread reconfigurations were put in place in healthcare settings to continue the provision of essential care. In high-income countries (HICs) hospitals provided only essential face-to-face appointments, strongly shifting delivery of care toward virtual forms (4). Widespread partner restrictions for pre-and post-natal appointments exacerbated feelings of isolation and loss. Women faced emergency settings alone and had to relay distressing information to their partners who were unable to accompany them (7). Additionally, racial disparities negatively impacted ethnic minority women during the birthing period, who purposefully sought out healthcare professionals (HCPs) who might better support them (8). Inconsistent regulations, both within and between countries, highlighted global care inequalities (9). As vaccination programmes commenced, concerns regarding vaccine safety arose due to rapid development and lack of information. The American College of Obstetricians and Gynecologists (ACOG) urged vaccination during pregnancy and breastfeeding to reduce disease risk and severity (10). The World Health Organization (WHO) stated the benefits outweighed any potential risks (11); however, the long-term effects on children born to mothers with or without COVID-19 vaccination remain unknown.

A systematic review of the women's experiences of maternity care, early in the pandemic, synthesized data from 31 global studies in both high-income (HICs) and low- and middle-income countries (LMICs), published between March 2020 and June 2021 (9). They found maternity care was negatively impacted by fast-changing policies, leaving pregnant women isolated and afraid. While isolation allowed bonding with newborns, this benefit was often overshadowed. Telehealth was only a limited substitute for face-to-face appointments, and partner restrictions were viewed negatively by all (9).

Our review sought to build on this work by extending the timeline of studies to the end of the pandemic, and beyond, to determine longer-term effects of the COVID-19 pandemic and associated service reconfigurations in order to inform future healthcare response to health-system shocks. This review evaluates women's experiences of maternity care reconfiguration during the COVID-19 pandemic in HICs, excluding the UK which was synthesized separately (12). Our team found that for women in the UK, a personalized an inclusive approach to care, along with clear and evidence-based communication to facilitate decision making were most important. This study forms of the wider RESILIENT programme of work on post-pandemic planning of maternity services (13, 14).

## 2 Methods

This review is registered with PROSPERO (CRD42022355948) and adheres to the PRISMA 2020 statement: an updated guideline for reporting systematic review guidelines (15) (see Supplementary Table S1).

### 2.1 Inclusion criteria

The SPIDER (Sample, Phenomenon of Interest, Design, Evaluation, and Research type) framework was utilized as was done in the original review (9).

The sample consisted of women planning pregnancy, pregnant or up to 6 months postpartum, and of any parity or risk status. The phenomenon of interest was maternity care experiences during the pandemic, focusing on labor, the postpartum period, and vaccinations across various healthcare settings, including hospitals, communities and homebirths. Study designs of interest included: published papers of qualitative research and mixed method studies where qualitative results could be extracted; survey designs with open-ended questions; qualitative descriptive and exploratory studies; ethnography; linguistic studies; and studies of public discourse. The evaluation of outcomes revolved around the narrative views and experiences of pregnant and up to 6-month postpartum women in HICs, excluding the UK, and their perspectives on maternity care during COVID-19. The research type included primary studies published between 01 June 2021 and 13 October 2022, updated to June 2024. The searches were limited to the English language.

### 2.2 Search strategy

**Figure 1:**
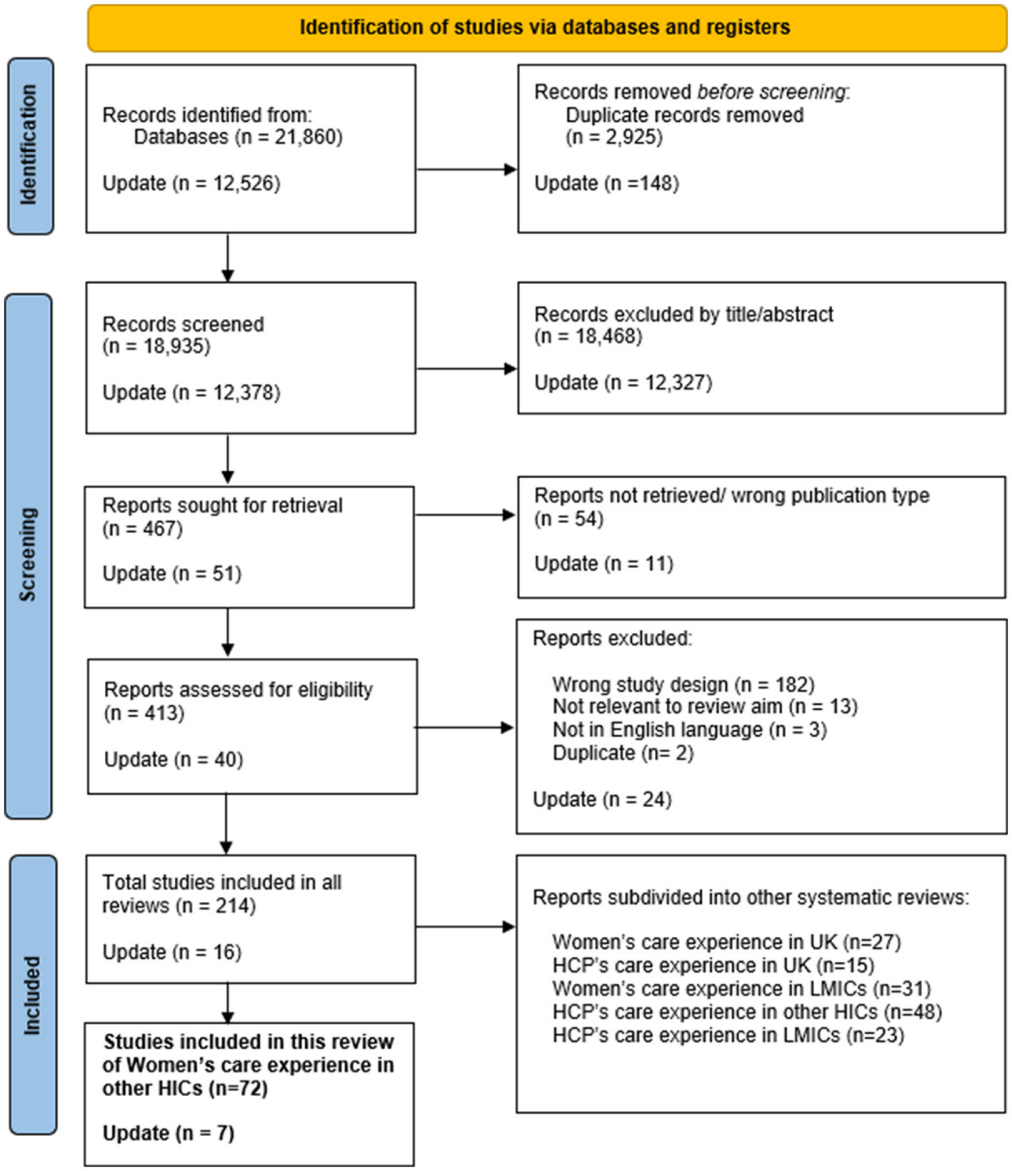
PRISMA flow chart of study selection process.

Systematic searches of electronic databases were carried out in: Scopus, MEDLINE, EMBASE, CINAHL, PsycINFO, and the Cochrane COVID Study Register. The search dates were 01 June 2021 to 13 October 2022, then further updated to June 2024. The search terms were formed on the basis of the SPIDER inclusion criteria (see Supplementary Table S2). The PRISMA flow chart presented in Figure 1 outlines the study selection process.

### 2.3 Study selection

Duplicate search results were removed using EndNote Reference Manager and uploaded to Rayyan (16), a systematic review web tool. Independent evaluation of titles and abstracts, followed by full-text reviews were conducted by a minimum of two reviewers of the RESILIENT study team at each stage. Differences in opinions during the review process were addressed in regular team meetings to maintain consistency and enhance the reliability of the evaluation process.

Given the large number of studies which met inclusion criteria, a decision was subsequently taken prior to data extraction, to divide the review by constituent population groups (i.e., women's or HCPs' experience) and geography based on where the study was conducted (i.e., in HICs, the UK, or rest of the world) to produce a series of more focused reviews. This decision was taken to manage the volume of retrieved studies, and to align the aims to the RESILIENT programme of work. This review relates to women's experiences in HICs; others have already been published (12, 17, 119). Studies outlining experiences of multiple participant groups or geographies were categorized into all relevant sub-reviews.

### 2.4 Quality assessment

In line with the review (9) to which this is an update, methodological quality of included studies was evaluated using an adapted version of an appraisal tool developed by The Evidence for Policy and Practice Information and Coordinating Center (18). This involves twelve criteria across three domains: study reporting quality, data collection and analysis reliability and validity, and study methods quality. Multiple reviewers independently assessed each study (CC, TD, ER, SML) and marked as *Yes, No*, or *Partial*. All studies were included regardless of quality to capture valuable experiential data; however, results were checked to ensure themes did not rely solely on low-quality papers.

### 2.5 Data extraction and synthesis

Multiple reviewers (CC, TD, ER, SML) extracted relevant information from each included study into a pre-designed Microsoft Excel spreadsheet. The data extracted included: study reference, aims, participant description, geography, study dates, data collection method, data analysis approach, and results themes identified by authors. Additionally, it was documented if the study evaluated any of the three principal thematic aims of the RESILIENT study—virtual care, self-monitoring, and vaccination.

Data synthesis was performed in the discussion sections of each paper, using NVivo (Lumivero, Denver, Colorado, USA) qualitative research software, to avoid redundant coding of identical themes from primary data and rendering logic circular. A slightly modified version of Thematic Synthesis (19) was employed. The RESILIENT study utilized five pre-determined concepts aligned with its aims: (1) Care-Seeking and Care Experience, (2) Ethical Future of Maternity Care, (3) Self-Monitoring, (4) Vaccination, and (5) Virtual Care, the latter three being key service reconfigurations during the pandemic. Data were initially grouped into these five pre-determined concepts and then themes and sub-themes derived inductively, by at least two reviewers independently (CC, TD, ER, SML) to maintain coherence of data synthesis. Regular meetings were held to discuss disagreements and agree on thematic results.

## 3 Results

### 3.1 Search and selection

The initial literature search identified 21,860 records, with no additional records found in register searches. After eliminating duplicates (*n* = 2,925), ineligible records based on title or abstract screening (*n* = 18,935), and inaccessible records (*n* = 54), 413 records underwent full-text review. After excluding additional ineligible records (*n* = 143), 201 records met the eligibility criteria. Of these, 72 studies examined women's maternity care experiences in HICs, excluding the UK, during the COVID-19 pandemic (20–91). An updated research to June 2024 found a further seven studies for inclusion, The PRISMA flow chart (Figure 1) summarizes this process (15).

### 3.2 Description of included studies

The characteristics and key findings of the 79 studies, with data from over 20,000 pregnant/postpartum women are presented in Supplementary Table S3. Alongside women's data, seven studies also included partners' experiences (34, 64, 73, 74, 85–87), eight included data on HCPs (21, 23, 42, 58, 71, 73, 77) and three included data from the UK (20, 32, 62), relevant data extracted as appropriate.

The most commonly addressed themes were Care Experience and Care Seeking (*n* = 73) (20–30, 32–39, 41–46, 48–62, 64, 66–90), Ethical Future of Maternity Care (*n* = 39) (20, 21, 23–26, 28–30, 35, 38, 39, 42, 45, 47–49, 54, 55, 57–63, 65, 68, 71, 72, 78, 81, 84, 87, 88, 90, 91) and Virtual Care (*n* = 32) (23–25, 30, 35, 37, 38, 41, 45, 48, 49, 55, 58–60, 62, 63, 65, 68, 73, 74, 76, 78, 81, 82, 84, 88). Fewer studies delved into Self-Monitoring (*n* = 3) (45, 69, 83) or COVID-19 Vaccination (*n* = 3) (47, 67, 91).

The data collection timeframe, based on all the papers synthesized within this review, spanned from September 2019 to February 2022 and was conducted primarily through semi-structured interviews exclusively (*n* = 45) (21, 23, 25, 26, 30, 31, 35, 37–39, 43, 45, 46, 48, 49, 52, 53, 55–57, 59, 60, 63, 65, 68–70, 73, 74, 76, 77, 79, 81–84, 86, 90, 91), online surveys featuring open-ended questions (*n* = 23) (22, 24, 29, 36, 40, 42, 44, 47, 50, 52, 54, 58, 61, 62, 64, 66, 67, 75, 85, 87–89), or a mixture of open-ended questions and semi-structured interviews (*n* = 6) (27, 28, 33, 41, 72, 78). Other methods of primary data included videos (*n* = 1) (20), chat forums (*n* = 1) (32), case study interviews (*n* = 1) (80), and social media posts written by parents (*n* = 1) (34), where formal qualitative data analysis had been conducted.

Thematic analysis (*n* = 59) (21–28, 30–36, 38–49, 52–54, 59, 63–65, 67–72, 74–77, 79, 80, 82–88, 91) was most commonly used for data analysis followed by content analysis (*n* = 10) (29, 37, 50, 51, 55, 58, 61, 62, 73, 89), interpretative phenomenological analysis (*n* = 3) (57, 60, 90), narrative analysis (*n* = 1) (20), constant comparative method (*n* = 1) (56), grounded theory (*n* = 1) (81), interpretive descriptive analysis (*n* = 1) (78) and natural language analysis (*n* = 1) (66).

### 3.3 Quality assessment

The quality of the included studies varied (see Supplementary Table S4). Twelve studies partially or fully satisfied ≤ 5/12 criteria (24, 30, 33, 40, 46, 53, 66, 67, 72, 75, 80, 82), 31 studies fulfilled between 6 and 9/12 criteria (20, 27, 28, 32, 34, 35, 39, 42, 45, 48, 50–52, 54, 56, 61, 63, 64, 68, 69, 74, 78, 79, 83, 84, 86, 87, 89), nine met 10/12 criteria (21, 31, 36, 47, 59, 77, 85, 88, 91), 21 met 11/12 criteria (22, 23, 25, 29, 37, 41, 43, 44, 49, 55, 57, 58, 60, 62, 73, 76, 81), and six met 12/12 criteria (26, 38, 65, 70, 71, 90). Common reasons for a reduced quality score included insufficient detail regarding patient and public involvement and inadequate descriptions of data collection and analysis. All studies were included for synthesis, however, integrity of synthetic themes were checked to ensure that removal of low-quality studies did not affect thematic results.

### 3.4 Synthesis and findings

Synthesis of included studies led to the development of 19 sub-themes across the five key concepts (Table 1). Supporting excerpts from included studies are presented in Tables 2–6.

**Table 1:** Process of theme development.

Resilient concept	Theme	Descriptive codes
Care-seeking and care experience	Communication with HCPs and care plans	Lack of communication and sensitivity. Positive forms of communication. Contradicting government information. How restrictions affected general maternal experiences and those in healthcare settings e.g. partner restrictions, and autonomy in care. Opportunity to change care experience.
	Exacerbating pre-pandemic difficulties.	Inequality in care. Safety and concerns for women and children. Maternal support.
	Maternal mental health and coping	The burden of the pandemic. Coping with mental health issues. Increased family bonding. Peaceful recovery and learning time. Reduced societal and familial pressures.
Virtual care	Access to virtual care	Digital divide. Increased convenience. Technical difficulties reduced access.
	Poor quality of care and insufficient support	Lack of physical exam induced fear and skepticism of the quality of care. Importance of the patient-HCP relationship and communication. Communicating with friends and family. Virtual communities and apps for social and educational support. Unsatisfied with antenatal classes. Reduces risk of infection.
	Preference for in-person care	Preference for the option of in-person care.
Self-monitoring	Equipment, capability, and implementation	Need to purchase own equipment leads to inequality in healthcare. Ability to use equipment. Heavy responsibility. Fear of identifying something wrong. Increased maternal pressure.
Vaccination	Vaccine education and external influences	Higher vaccine knowledge influenced vaccine uptake positively. External influences have a negative effect on vaccine uptake. Wanting to protect their family.
	Trust in research and professionals	Vaccine uptake is influenced by HCP opinion. Trust in HCP increases vaccine uptake. Concern over-vaccination harming the fetus. The importance of understanding scientific evidence. Gestational age influenced vaccine hesitancy.
Ethical future of maternity care services	Future implementation of virtual care	Making use of wider resources to aid with quality of care. Better access to healthcare professionals in the community. Providing incentives to attend appointments. Improved access to psychological healthcare. Assessing barriers to virtual care and vaccination.
	Equitable patient-centered care	Improving communication and autonomy. Social support. Exposure to racial problems in the hospital.

**Table 2:** Concept 1: care-seeking and care experience.

Sub-themes	Excerpts
Communication with HCPs and care plans	“They had limited knowledge about what to expect, or what or who would be available for them, either during antenatal check-ups or at the place they were going to give birth.” (44) “The findings of our study show that postnatal care was experienced as busy, cold, and lacking compassion. Women reported feeling like a bother when they asked for help and felt that the postnatal wards were critically understaffed.” (44) “The pandemic also changed practice in regard to birth-related interventions. For example, no clinical evidence suggested that the projected volume of COVID cases was a medically necessary indication for pregnancy interventions, yet many in our study reported this practice. These interventions included mandatory epidurals, inductions, cesarean sections, and stripping membranes. It is clear that individuals in our study felt more empowered when they participated in the decision to intervene and felt violated when they could not.” (36) “Concerning mother-infant contact after childbirth, it was often allowed only after a negative swab result, even though in May 2020 it was established that being a COVID-19 positive woman is not a valid reason for mother-child separation.” (34)
Exacerbating pre-pandemic difficulties	“We found that intrapartum care was similar to pre-pandemic care, including rates of cesarean birth; and unfortunately, disparities persisted with rates of preterm births and less respectful maternity care for Black respondents compared with White respondents.” (29) “By restricting visitors, many BIPOC patients had their support networks disrupted, reducing sources of advocacy and protection from racism and mistreatment in the hospital setting. In addition, nurse participants highlighted how these policies were biased in nature and likely contributed to furthering health disparities in BIPOC communities.” (21) “Participants in the current study indicate that pandemic-induced policies in maternal health care institutions have continued… Sitting in their own urine while in pain, crying alone for hours, being yelled at while trying to care for a sick newborn just days after abdominal surgery; this is harsh and undignified treatment, and this study shows that these are unintended outcomes of policies that limit the presence of a support person during the postpartum period.” (68) “The women also expressed concerns about the quality of care that would be provided in case their baby was tested positive; questions about the nature of the treatment, possible hospitalization, medication and side effects, and generally about the management of the virus were commonly voiced. Questions about both short-term and long-term consequences of the virus on their baby's health and development were also raised.” (54)
Maternal mental health and coping	“Feelings of fear, uncertainty, and loss were unique to the pandemic, adding layers of psychological stress to an already emotional time, and pandemic-related employment concerns contributed to economic stress. For some participants, self-care, interpersonal, and structural support helped alleviate those stressors. Effective self-care strategies and interpersonal supports included taking walks and receiving virtual support and advice from loved ones.” (37) “Women commented on the lack of information or support for ‘baby blues' or other issues related to mood. For other women, the lack of access to breastfeeding consultations or other postnatal support had a significant effect on their perceived ability to care for their baby and consequently, on their overall wellbeing.” (54) “The most commonly mentioned positive element from postnatal women was having fewer visitors in the hospital. Women in our study reported appreciating the time to bond with their neonate, improved opportunity to initiate breastfeeding, increased attention from midwives, and relief from the pressure to socialize immediately after birth.” (58)

#### 3.4.1 Concept 1: care-seeking and care experience

This concept included 73 studies (20–30, 32–39, 41–46, 48–62, 64, 66–90) producing three themes: (1) Changes to communication with HCPs and care plans, (2) Exacerbating pre-pandemic difficulties, and (3) Maternal mental health and coping (Table 2).

##### 3.4.1.1 Changes to communication with HCPs and care plans

Communication challenges between expectant mothers, HCPs, and governments were highlighted, with women uncertain about antenatal procedures (44) due to varying guidance between countries' governments (56). Efforts such as virtual Q&As and personalized care were made (82), but postnatal care often felt busy and impersonal, causing women to hesitate in seeking help for fear of burdening the staff (44).

COVID-19 drastically changed maternity care, disrupting expectant parents' plans with restrictions on birthing partners and limited newborn visits, causing disappointment and anxiety (32). Changes in birth-related interventions, often without clear medical necessity, underscored the need for participant involvement in decision-making to ensure respect for patient autonomy (36). Hospital policies such as early discharge and visitor limitations increased anxiety and isolation (29), and some hospitals required negative COVID-19 tests before mother-newborn contact, leading to prolonged mother-infant separation (34).

##### 3.4.1.2 Exacerbating pre-pandemic difficulties

The pandemic exacerbated disparities in maternal healthcare. Black, Indigenous, and People of Color (BIPoC) women faced less respectful care compared to White women (21). Policies intensified existing inequities, leading to negative experiences and a lack of support (68). Increased anxiety over COVID-19 risks and care quality for both mothers and infants were reported, including the unknown long-term effects of the vaccine (54, 68). Women emphasized the need for individualized care from known professionals and support persons to improve provider-patient relationships (35, 84).

##### 3.4.1.3 Maternal mental health and coping

As global restrictions shifted, mothers experienced heightened fear and anxiety during the perinatal period due to uncertainty (30). Post-hospital discharge, women faced challenges such as inadequate support for mood-related issues and limited postnatal care (54). Despite these difficulties, participants engaged in self-care activities, including seeking outdoor spaces, virtual support, and healthy coping strategies (57). The pandemic created conditions that reshaped daily life. Remote work allowed mothers to be more present with their infants, making breastfeeding more manageable (34). The ability to provide full-time infant care strengthened parental connections, while limited hospital visitors provided a quieter, less stressful postpartum environment (58).

#### 3.4.2 Concept 2: virtual care

This was conceptualized as a broad concept of remote care delivery, covering medical consultations by telephone or video, as well as virtual care and social support via apps, chat forums, or social media. It included 32 papers (23–25, 30, 35, 37, 38, 41, 45, 48, 49, 55, 58–60, 62, 63, 65, 68, 73, 74, 76, 78, 81, 82, 84, 88) producing three descriptive themes: (1) Access to virtual care, (2) Poor quality of care and insufficient support, and (3) Preference for in-person care (Table 3).

**Table 3:** Concept 2: virtual care.

Themes	Excerpts
Access to virtual care	“The pandemic amplified the digital divide noted among populations who could not access the technology or internet to utilize telehealth or other internet-based modalities to support communication.” (35) “Many participants in this study supported the use of telehealth in their prenatal care, citing reasons, including a sense of greater convenience, less time commitment, and less frustration from logistic steps of navigating the clinical setting compared to in-person visits.” (37) “For some patients, technical difficulties and navigating the software were sources of stress in their telemedicine experience, specifically for Spanish-speaking mothers” (63)
Poor quality of care and insufficient support	“Participants expressed concern over receiving suboptimal care via telemedicine, leading to anxiety over the health of their children.” (49) “A key aspect of concern to the reduced access to care and implementation of telehealth was the lack of physical assessment. In our study, the inability to have a physical assessment concerned women, causing them to feel a heightened sense of responsibility about their and their baby's health.” (84) “Challenges within the healthcare system during the pandemic included feeling unsupported via telehealth” (37) “In addition, they valued telehealth as a way to avoid possible exposure to COVID-19 and the potential negative consequences of infection for themselves, the pregnancy, and their family.” (37) “Finally, our data indicate that lack of support was a factor in many women's inability to breastfeed successfully. Although some were able to access online supports, these were uniformly viewed as ineffective.” (68)
Preference for in-person care	“Our findings confirm that women had difficulty navigating the restructured health visitor support with online/telephone contacts. We found that a barrier for contacting healthcare providers was that the women found it difficult to describe their worries without face-to-face contact, especially when these worries concerned motherhood adaptation, the infant's social progression and everyday routines.” (55) “While women in our cohort found the increased access to telehealth to be very positive, many of them provided caveats to their endorsement of the practice. These were chiefly related to wanting telehealth to be offered as an option rather than a mandate.” (58)

##### 3.4.2.1 Access to virtual care

During the pandemic, online consultations worsened inequalities related to access to technology and internet connectivity (35). Technical difficulties, particularly for Spanish-speaking mothers in the USA, were a concern (63). Despite these challenges, telehealth was favored on occasion for its convenience (37).

##### 3.4.2.2 Poor quality of care and insufficient support

The absence of physical examinations during virtual care emerged as a significant worry for women, as it amplified their sense of responsibility for their own and their baby's health (84). The heightened anxiety extended to the wellbeing of their other children (49). Women felt unsupported by telehealth, with insufficient physician assistance and ineffective online resources impacting breastfeeding success (68). These increased feelings of isolation and made accessing reliable breastfeeding information harder (78). Despite this, telehealth was appreciated for reducing COVID-19 exposure risks (37).

##### 3.4.2.3 Preference for in-person care

Participants valued expanded telehealth but stressed it should be optional (58). For more sensitive topics such as mental health problems, many preferred in-person consultations to establish a meaningful physician-patient relationship, finding it difficult to communicate concerns without face-to-face interactions (55, 63).

#### 3.4.3 Concept 3: self-monitoring

This concept, presented in only three studies (45, 69, 83), focused on using equipment like Doppler scans and blood pressure machines for pregnancy monitoring at home. Due to limited data, one theme was derived: Equipment, capability, and implementation (Table 4).

**Table 4:** Concept 3: self-monitoring.

Themes	Excerpts
Equipment, capability, and implementation	“During the pandemic, self-monitoring equipment such as blood pressure machines were provided in some areas of the United Kingdom … whilst in some areas of America (as with our study participants) it was recommended women purchase these …” (83) “However, very few participants expressed feelings of empowerment and control, and troublingly, the stakes of effectively exercising responsibility over childbirth are highest for the people who need the most care. Moreover, pregnant people are expected to take on additional responsibility for their health, the health of their babies, and the outcome of their birth while their access to clinical and psychosocial birth supports has been reduced.” (69) “Another concern pertained to a fear that they would be the ones to directly identify miscarriage or fetal loss by detecting the lack of a heartbeat using the fetal Doppler.” (45)

##### 3.4.3.1 Equipment, capability, and implementation

Access to equipment varied between groups, worsening existing inequalities. In countries such as the USA, patients had to purchase their own home monitoring devices, creating care disparities based on socioeconomic status (83). Home monitoring equipment like fetal Dopplers raised concerns about users' ability to operate them effectively. Reduced clinical support in teaching women how to use such devices, placed more responsibility on pregnant individuals, increasing feelings of unease, especially for mothers requiring additional care (45).

#### 3.4.4 Concept 4: vaccination

This concept was reported by only three studies (47, 67, 91) and two themes were derived: (1) Vaccine education and external influences, (2) Trust in research and professionals (Table 5).

**Table 5:** Concept 4: vaccination.

Themes	Excerpts
Vaccine education and external influences	“Women in this study were strongly influenced by their support network, and most of the time this influence prevented them from being vaccinated.” (91) “There were many influences that impacted decisions to vaccinate. One commonly found influence in this study and others is the desire to protect themselves and/or their infant from infectious diseases.” (91)
Trust in research and professionals	“In our study, we found participants were more interested in the COVID-19 vaccine if they received a recommendation from their provider or assurance about the vaccine.” (67) “Safety of their unborn infant was the primary concern of women, regardless of stated likelihood of vaccine receipt.” (47) “Concerns about long-term effects, and general anxiety around how new the vaccine is were more commonly cited by women with a lower likelihood of vaccine receipt. Whereas those with higher stated likelihood of COVID-19 vaccine receipt spoke more about demonstrating safety through data.” (47)

##### 3.4.4.1 Vaccine education and external influences

Greater knowledge about vaccines increased uptake due to a sense of trust in vaccinations and science in general. However, peer influence and misunderstandings about vaccine function often deterred acceptance. Some women rejected vaccines, believing their current lack of ill-health meant they would not be severely affected by COVID-19 (91). Vaccination decisions were driven by the desire to protect both mother and infant from diseases and to prioritize both immediate and long-term health by ensuring a safer environment (91).

##### 3.4.4.2 Trust in research and professionals

HCP recommendations were crucial for vaccine uptake; participants were more likely to vaccinate if their HCP supported it (67). Trust in providers improved patient perceptions of vaccine effectiveness and safety. Clear guidance on vaccination and accessibility during pregnancy was vital for positive vaccination decisions (47). Concerns about long-term effects of the vaccine and a lack of short-term safety data specifically for pregnancy increased anxiety and resistance, leading women to request more scientific proof of safety (47).

#### 3.4.5 Concept 5: ethical future of maternity care services

There were 39 studies (20, 21, 23–26, 28–30, 35, 38, 39, 42, 45, 47–49, 54, 55, 57–63, 65, 68, 71, 72, 78, 81, 84, 87, 88, 90, 91) which addressed the final concept of imaging a better future for maternity care in a post-pandemic world and produced two themes: (1) Future implementation of virtual care, (2) Equitable patient-centered care (Table 6).

**Table 6:** Concept 5: ethical future of maternity care services.

Themes	Excerpts
Future implementation of virtual care	“Based on the benefits described by mothers of both visit types in our study, we advocate for making both in-person and video visits available options for all mothers.” (48) “In addition, online resources for women on hospital-specific websites, including virtual tours and online classroom perinatal education, may help offset preparedness stress.” (25) “However, hospitals must proactively evaluate patient-level barriers to successful implementation of telemedicine. For example, a patient's ability to successfully complete a telemedicine visit varies based on socioeconomic background, geographic location, digital and health literacy, and English language proficiency.” (63) “Facilitate easier access to perinatal-trained mental health workers, such as using navigators to assist women to connect with an appropriate provider.” (57)
Equitable patient-centered care	“To ensure good quality of maternity care it is critical to have strong communication applications between health professionals and women to reinforce continuity in the care.” (88) “While the perceived benefit from visitor restrictions includes a reduced potential for exposure to the COVID-19 virus, providers and health care systems need to balance the ethical quandary of then increasing risk for perpetuating health disparities, particularly exposure to racism and mistreatment by providers for BIPOC communities.” (21) “Prioritize clear communication regarding postpartum care expectations and how to access telephone and/or in-person breastfeeding social support that follows Public Health guidelines.” (78)

##### 3.4.5.1 Future implementation of virtual care

It is essential to offer diverse care options to advance maternal care. Extending care to include both in-person and virtual services is crucial for improving accessibility (48). Suggestions such as providing virtual hospital tours were highlighted as a way to ease anxiety about entering an unfamiliar environment (25). Barriers to virtual care must be addressed, namely, socioeconomic factors, digital literacy and language proficiency to ensure equitable access (63). Enhancing vaccination accessibility is essential for improving uptake and ensuring mother and infant safety (91). A large demand for mental health services means strategies to expedite communication between mothers and HCPs are necessary (57).

##### 3.4.5.2 Equitable patient-centered care

Autonomy in care is crucial, enabling patients to have control over their treatment and the decisions made by HCPs (58, 78, 88). Minority ethnic groups faced health disparities and disrespectful treatment, especially with visitor restrictions taking away support persons (21). Maintaining high-quality maternity services requires improved communication between HCPs and women, including services such as online support for maternal wellbeing (78, 88).

## 4 Discussion

We present the results of a large and expansive systematic review of women's maternity care experiences during the COVID-19 pandemic in HICs around the world, excluding the UK which has been synthesized separately (12). We expand on the Flaherty et al. review by adding 79 new studies published between June 2021 and June 2024, the former presenting data on 14 studies on women from HICs, and 31 globally. The review by Flaherty et al. found five themes about women's experiences of maternity care during the pandemic: Altered maternity care; COVID-related restrictions; Infection prevention and risk; “The lived reality”—navigating support systems; and Interactions with maternity services. Our findings support these narratives and add to it by synthesizing additional information on COVID-19 vaccinations for pregnant women, and reflections on a more equitable future for maternity care globally.

### 4.1 Main findings and comparison with the literature

In relation to care-seeking and care-experience, this review demonstrated the critical importance of consistent communication in building trust and encouraging attendance at healthcare services during crises. Confusing guidance and fear for safety led women to delay antenatal scans for up to 4 months (92). Similar to the original review (9) and our findings from the UK (12), this review also showed extreme dissatisfaction over partner restrictions during antenatal care appointments and labor and delivery, which led to feelings of separation and exclusion (32). Delays in mother-newborn contact due to pending negative COVID-19 results and disapproval of mother-infant separation were common (34).

Data from Italian and Spanish women synthesized as part of our review reported feeling violated by unwanted interventions such as mandatory epidurals, inductions and cesarean sections, highlighting the need for patient autonomy (36). However, an Australian study reported a reduction in such interventions, suggesting varying approaches to maternity care across countries (93). Perinatal mental health emerged as a critical theme, with mothers expressing fears for their children, themselves, and their families. This has been observed in the UK (12), and in LMICs (94) where mothers reported increased anxiety during in-person appointments and increased isolation and loneliness (19). Women resorted to a range of coping strategies to manage the challenging consequences of disrupted care and reduced support systems, similar to data reported in other global syntheses of the impact of COVID-19 on maternity care (95). These findings should also be considered within the broader context of COVID-19 infection experiences, wherein psychological distress, uncertainty, and unmet healthcare needs have been showed to have persisted beyond care discharge, including the context of long COVID. This suggests that the fear and uncertainty reported by perinatal women reflect wider systemic gaps in continuity of care, access to appropriate support, and responsiveness of health services (96).

Our findings that women hesitated to seek help due to concerns about burdening healthcare professionals can be further understood through the lens of help-seeking behavior frameworks. These emphasize that care-seeking is not a singular action but a multi-stage process involving symptom appraisal, interpretation of severity, and decisions when and whether to disclose concerns (97). During the pandemic, disruptions to care pathways and perceptions of overstretched services may have altered women's thresholds for seeking support, leading to delayed disclosure or normalization of potentially concerning symptoms.

The widespread shift to telehealth services globally was associated with both benefits and challenges. This transition emphasized the digital divide, with lower socio-economic populations struggling to access devices and internet connections. In a study with HCPs in the USA 78% identified service users' access to technology as the biggest operational barrier (98). While telehealth offered convenience and flexibility, concerns emerged about the quality of care provided, seen also in the UK (12). Similarly, both in the UK and other HICs, women feared the lack of physical assessments could result in overlooked problems, heightening anxiety as they shouldered more health-related responsibilities; a finding echoed by both HCPs and women in other work (99).

Although our review highlights a preference for in-person breastfeeding support, an international study found mothers with access to virtual support groups were 80% more likely to breastfeed compared to those without such support (100). On the other hand, for women with complex care needs, our results are in line with other research. Primiparous women, particularly those with pre-existing comorbidities, preferred in-person care for its comprehensive attention (101, 102). Other authors found that although 61.4% of mothers were satisfied with telehealth, 93.2% would have opted for in-person care to reduce uncertainty (101).

Moreover, it would be prudent to consider the workforce impact of implementing digital modalities more expansively. Increased workload for already stretched maternity workforce, usability by staff without adequate training, misalignment of new digital applications with existing clinical IT systems, and overall lack of digital infrastructure pose barriers to implementation that need to be given sufficient consideration (17, 103, 104).

One study highlighted concerns among women in the USA about obtaining monitoring equipment not provided by their HCPs. In contrast, in the UK, maternity units provided free blood pressure monitors (105), revealing differences in care based on socioeconomic backgrounds and healthcare models. A survey in the USA, found 45.2% of women who partook in virtual care were asked to self-monitor, with success influenced by insurance type, income, education, and age, highlighting implementation problems in lower-income populations (106). We found women were overly burdened and remained uncertain about performing complex at-home monitoring, such as fetal heart rate monitoring which should be done under physician supervision to reduce errors, as echoed by participants in the Czech Republic (107) and Indonesia (108).

Despite COVID-19 vaccination being readily available and recommended for use in most HICs, hesitancy persisted, particularly among marginalized populations. Among New Zealand's Indigenous communities, hesitancy was linked to cultural beliefs and a lack of perceived need for vaccination (91). Similarly, a UK study highlighted multiple factors influencing vaccine uptake, including beliefs that the vaccine was unimportant, conspiracy theories, suspicion over quick manufacturing, and distrust in the healthcare system (109), which was reflected in our UK review (12). Socioeconomic factors have been found to be associated with vaccine acceptance, with a multinational study revealing that women with lower education levels were 42% less likely to accept a COVID-19 vaccine, and unemployed women were 29% less likely to accept the vaccine (110). These findings emphasize the intersectionality of socioeconomic factors, education and vaccine attitudes, indicating the need for tailored strategies and education to promote vaccination equity. Trust in and relationship with HCPs was a critical factor for vaccine uptake in this review. An Italian survey discovered that 58% of women who did not receive a flu vaccine during pregnancy did so based on a lack of HCP recommendations (111). Similarly, a Canadian study reported increased confidence in COVID-19 vaccines after discussing safety concerns with HCPs (112).

Finally, our review synthesized data reflecting on building an ethical and equitable future for maternity services. Infection prevention protocols and hospital policies, such as restrictions placed on partners or support persons, led to women—particularly marginalized women—being deprived of who could advocate for them. Communication hurdles stemming from use of protective equipment such as face masks impacted women with hearing impairments; the inability to lip-read led to inadequate care, and highlighting the need for more inclusive communication strategies (113). Over-reliance on technology during the pandemic, and in particular lockdown periods, led to the exclusion of some groups. Our review identified a need for improving virtual care services in the future by addressing socio-economic, geographical, digital literacy, and language barriers. In the UK (12), we called for a personalized approach to delivering virtual maternity care, where individual needs were assessed first. A Swedish study evaluated a medical app available to women at all hours of the day, finding that while accessibility improved, registration requirements such as bank identification excluded some users (114).

Patient autonomy and empowerment need to be held in focus in future planning of maternity services (58, 78, 88). Concerns for infant safety were prevalent in this review, with an Australian study highlighting that parents were often not involved in their newborn's health decisions (115), potentially increasing worries and emphasizing the need for better oversight and accountability in care settings. A Canadian study echoes the importance of collaborative decision-making, with 95.2% of participants stating a need for increased autonomy and not being coerced or pressured into accepting interventions (116) which was also reflected in our review of women's experiences in the UK (12), wherein improved production and dissemination of information was highlighted as a future priority. Delivery of equitable, patient-centered care is closely linked to organizational culture, staff psychological wellbeing and positive safety attitudes; critical for ensuring patient safety. Without prioritization of safety culture and staff wellbeing, efforts to implement equitable care may be undermined by burnout and attrition (117).

### 4.2 Strengths, limitations, and future research

A key strength of this review is its focus on women in HICs, enhancing the specificity of our findings. Our inclusion of 79 studies provides an expansive overview of data from several countries with broadly similar health systems and resource availability. Limitations of the review include the inclusion of only English-language papers, which may have limited our scope. This decision was made to remain consistent with our other complimentary reviews. However, given the focus on HICs of which the majority of studies were from English speaking countries, namely USA, Canada, and Australia, we do not expect many studies to have been excluded by this criteria. We also omitted research related to contraception and abortion services, due to the variable nature of how these services were handled during the pandemic in different HICs. While we included 12 studies which met 5 or less of the 12 criteria in the quality assessment tool; this was an assessment of the methodological quality only, in particular of reporting and transparency. As we synthesized discussion sections of included papers, we hypothesized that poor reporting practice in these papers, would not greatly impact our study results. In a sensitivity analysis, wherein the low-quality papers were removed from the synthesis; we assessed the integrity of derived themes to ensure they are not affected, which was found to be true. Data from these studies do not solely contribute to any of our themes or concepts, and as such inclusion of them has not altered the results, regardless of study quality. Additionally, since our synthesis relied on discussion sections rather than raw participant data, it may have introduced an additional interpretive layer through the lens of the original authors. While methodologically pragmatic for managing a large synthesis, this approach may limit the depth and nuance of findings, particularly for intersectional, culturally specific, or marginalized experiences. The inclusion of qualitative data from a wide range of study types, including surveys and mixed-methods papers, meant the synthesis was primarily aggregative and may not have fully captured the contextual or structural factors underlying women's experiences. This limited our ability to theorize variations across settings or propose comprehensive conceptual frameworks. Future research is necessary to contextualize these differences, including on the impact of health system shocks on postnatal and infant care, which tends to be decentralized, and community based. A structured approach should be used to map digital intervention implementation, such as using implementation science frameworks, to ensure feasibility, effectiveness and equitability (118). Additionally, studies should explore improving self-monitoring at home and comparing care provision between HICs and LMICs to identify disparities and areas for improvement.

## 5 Conclusion

To strengthen maternity care during future health crises in HICs, we recommend the following be considered:

(1) Enhance access to essential services

- Improve telehealth infrastructure to ensure equitable and reliable virtual care.
- Expand vaccination programmes by increasing accessibility, streamlining distribution, and addressing hesitancy through public education.
- Strengthen mental health services by increasing accessibility and raising awareness of available support.

(2) Support self-monitoring for pregnant women

- Subsidize self-monitoring equipment, particularly for underserved communities.
- Provide clear guidance and training on at-home monitoring of maternal and fetal health.

(3) Strengthen patient autonomy and hospital policies

- Ensure collaborative decision-making and transparent communication between healthcare providers and patients.
- Improve hospital policies to allow greater partner inclusion in maternity care and postpartum support.
- Prioritize skin-to-skin contact and rooming-in, to support infant bonding and wellbeing.

## Data Availability

The original contributions presented in the study are included in the article/Supplementary material, further inquiries can be directed to the corresponding author.

## The RESILIENT Study Group consists of

*Chief Investigator:* Prof. Laura A. Magee (King's College London); and *Co-Investigators:* Prof. Debra E. Bick (The University of Warwick), Dr. Harriet Boulding (King's College London), Dr. Kathryn Dalrymple (King's College London), Ms. Tisha Dasgupta (King's College London), Prof. Emma L. Duncan (King's College London), Dr. Abigail Easter (King's College London), Prof. Julia Fox-Rushby (King's College London), Miss. Gillian Horgan (King's College London), Prof. Asma Khalil (St. George's University Hospitals NHS Foundation Trust & Liverpool Women's NHS Foundation Trust), Ms. Alice McGreevy (King's College London), Dr. Hiten D. Mistry (King's College London), Prof. Eugene C. Nelson (Dartmouth College), Prof. Lucilla Poston (King's College London), Mr. Paul Seed (King's College London), Dr. Sergio A. Silverio (King's College London & University of Liverpool), Dr. Marina Soley-Bori (King's College London), Dr. Florence Tydeman (King's College London), Ms. Aricca D. Van Citters (Dartmouth College), Dr. Sara White (King's College London), Prof. Ingrid Wolfe (King's College London), Prof. Yanzhong Wang (King's College London), & Prof. Peter von Dadelszen (King's College London).
