# Supplementary material for: Women's experiences of maternity care in high-income countries during the pandemic health system shock: a follow-up systematic review and qualitative evidence synthesis
Source: Front Public Health. 2026 Apr 22;14:1715725. doi: 10.3389/fpubh.2026.1715725 (PMC13143912; doi:10.3389/fpubh.2026.1715725)
Supplement: Supplementary file 1 [file Table_1.docx]

# Supplementary Information

| **Table No.** | **Description** | **Page No.** |
| --- | --- | --- |
| S1 | PRISMA Reporting checklist | 2 |
| S2 | Search strategy | 5 |
| S3 | Description of included studies | 6 |
| S4 | Critical appraisal of studies | 31 |

### Table S1: PRISMA reporting checklist

| **Section and Topic** | **Item #** | **Checklist item** | **Page where item is reported** |
| --- | --- | --- | --- |
| **TITLE** | | |  |
| Title | 1 | Identify the report as a systematic review. | 1 |
| **ABSTRACT** | | |  |
| Abstract | 2 | See the PRISMA 2020 for Abstracts checklist. | 2 |
| **INTRODUCTION** | | |  |
| Rationale | 3 | Describe the rationale for the review in the context of existing knowledge. | 4 |
| Objectives | 4 | Provide an explicit statement of the objective(s) or question(s) the review addresses. | 5 |
| **METHODS** | | |  |
| Eligibility criteria | 5 | Specify the inclusion and exclusion criteria for the review and how studies were grouped for the syntheses. | 6 |
| Information sources | 6 | Specify all databases, registers, websites, organisations, reference lists and other sources searched or consulted to identify studies. Specify the date when each source was last searched or consulted. | 6 |
| Search strategy | 7 | Present the full search strategies for all databases, registers and websites, including any filters and limits used. | 6, Table S2 |
| Selection process | 8 | Specify the methods used to decide whether a study met the inclusion criteria of the review, including how many reviewers screened each record and each report retrieved, whether they worked independently, and if applicable, details of automation tools used in the process. | 6-7 |
| Data collection process | 9 | Specify the methods used to collect data from reports, including how many reviewers collected data from each report, whether they worked independently, any processes for obtaining or confirming data from study investigators, and if applicable, details of automation tools used in the process. | 7, Table S3 |
| Data items | 10a | List and define all outcomes for which data were sought. Specify whether all results that were compatible with each outcome domain in each study were sought (e.g. for all measures, time points, analyses), and if not, the methods used to decide which results to collect. | 7, Table S3 |
|  | 10b | List and define all other variables for which data were sought (e.g. participant and intervention characteristics, funding sources). Describe any assumptions made about any missing or unclear information. | 7, Table S3 |
| Study risk of bias assessment | 11 | Specify the methods used to assess risk of bias in the included studies, including details of the tool(s) used, how many reviewers assessed each study and whether they worked independently, and if applicable, details of automation tools used in the process. | 7 |
| Effect measures | 12 | Specify for each outcome the effect measure(s) (e.g. risk ratio, mean difference) used in the synthesis or presentation of results. | n/a |
| Synthesis methods | 13a | Describe the processes used to decide which studies were eligible for each synthesis (e.g. tabulating the study intervention characteristics and comparing against the planned groups for each synthesis (item #5)). | 7 |
|  | 13b | Describe any methods required to prepare the data for presentation or synthesis, such as handling of missing summary statistics, or data conversions. | n/a |
|  | 13c | Describe any methods used to tabulate or visually display results of individual studies and syntheses. | n/a |
|  | 13d | Describe any methods used to synthesize results and provide a rationale for the choice(s). If meta-analysis was performed, describe the model(s), method(s) to identify the presence and extent of statistical heterogeneity, and software package(s) used. | 7 |
|  | 13e | Describe any methods used to explore possible causes of heterogeneity among study results (e.g. subgroup analysis, meta-regression). | n/a |
|  | 13f | Describe any sensitivity analyses conducted to assess robustness of the synthesized results. | n/a |
| Reporting bias assessment | 14 | Describe any methods used to assess risk of bias due to missing results in a synthesis (arising from reporting biases). | n/a |
| Certainty assessment | 15 | Describe any methods used to assess certainty (or confidence) in the body of evidence for an outcome. | n/a |
| **RESULTS** | | |  |
| Study selection | 16a | Describe the results of the search and selection process, from the number of records identified in the search to the number of studies included in the review, ideally using a flow diagram. | 8, Figure 1 |
|  | 16b | Cite studies that might appear to meet the inclusion criteria, but which were excluded, and explain why they were excluded. | Figure 1 |
| Study characteristics | 17 | Cite each included study and present its characteristics. | 9-10, Table S3 |
| Risk of bias in studies | 18 | Present assessments of risk of bias for each included study. | 10, Table S4 |
| Results of individual studies | 19 | For all outcomes, present, for each study: (a) summary statistics for each group (where appropriate) and (b) an effect estimate and its precision (e.g. confidence/credible interval), ideally using structured tables or plots. | 9-10, Table S3 |
| Results of syntheses | 20a | For each synthesis, briefly summarise the characteristics and risk of bias among contributing studies. | Table S3 |
|  | 20b | Present results of all statistical syntheses conducted. If meta-analysis was done, present for each the summary estimate and its precision (e.g. confidence/credible interval) and measures of statistical heterogeneity. If comparing groups, describe the direction of the effect. | 10-17 |
|  | 20c | Present results of all investigations of possible causes of heterogeneity among study results. | n/a |
|  | 20d | Present results of all sensitivity analyses conducted to assess the robustness of the synthesized results. | n/a |
| Reporting biases | 21 | Present assessments of risk of bias due to missing results (arising from reporting biases) for each synthesis assessed. | 10, n/a |
| Certainty of evidence | 22 | Present assessments of certainty (or confidence) in the body of evidence for each outcome assessed. | 10, n/a |
| **DISCUSSION** | | |  |
| Discussion | 23a | Provide a general interpretation of the results in the context of other evidence. | 18-20 |
|  | 23b | Discuss any limitations of the evidence included in the review. | 20 |
|  | 23c | Discuss any limitations of the review processes used. | 20 |
|  | 23d | Discuss implications of the results for practice, policy, and future research. | 21 |
| **OTHER INFORMATION** | | |  |
| Registration and protocol | 24a | Provide registration information for the review, including register name and registration number, or state that the review was not registered. | 6 |
|  | 24b | Indicate where the review protocol can be accessed, or state that a protocol was not prepared. | n/a |
|  | 24c | Describe and explain any amendments to information provided at registration or in the protocol. | n/a |
| Support | 25 | Describe sources of financial or non-financial support for the review, and the role of the funders or sponsors in the review. | 22 |
| Competing interests | 26 | Declare any competing interests of review authors. | 23 |
| Availability of data, code and other materials | 27 | Report which of the following are publicly available and where they can be found: template data collection forms; data extracted from included studies; data used for all analyses; analytic code; any other materials used in the review. | Supplementary tables |

### Table S2: Search strategy

| **Concept** | **SEARCH TERMS** |
| --- | --- |
| Sample | mother OR woman OR women OR midwives OR midwife* OR nurse* OR clinician OR physician OR doctor OR obstetric* OR professional |
|  | **AND** |
| Phenomenon of Interest | (maternity ADJ care) OR healthcare OR ‘health- care’ OR matern* OR birth* OR childbirth OR pre- nan* OR labour OR labor OR antenatal OR antepar- tum OR postnatal OR postpartum OR post-partum OR puerperium AND coronavirus* OR corona virus* OR COVID-19 OR COVID OR covid OR Covid2019 OR SARS-CoV* OR SARSCov* OR new CoV* OR novel CoV* |
|  | **AND** |
| Study Design and Evaluation | experiences OR experience OR view* OR perceptions OR perception OR voices OR narratives OR qualitative OR (mixed ADJ method) OR ‘grounded theory’ OR phenomenology OR ‘action research’ |

### **Table S3**: Characteristics of included studies

| **Ref** | **Country** | **Study aim** | **Description of participants** | **Dates when study was conducted** | **Data collection method** | **Data analysis method** | **Themes identified** | **Core RESILIENT aims** | | |
| --- | --- | --- | --- | --- | --- | --- | --- | --- | --- | --- |
|  |  |  |  |  |  |  |  | **Virtual care** | **Self-Monitoring** | **Vaccination** |
| Ajayi, MCN, 2021. | USA (n=60), **UK (n=8)**, Canada (n=6), Australia (n=2), Spain (n=1), Jamaica (n=1), Philippines (n=1), Switzerland (n=1), Not reported (n=3). | To explore childbearing experiences of new mothers in the context of Covid-19 and to assess what mothers feel that health care workers could have done differently in response to Covid-19. | N=83 Women | Jan 2020 – Sepr 2020 | Video recording | Narrative analysis | 1: Sense of loss 2: Hospital experience: response to pandemic. 3: Experience with health care professionals: 4: Overall birth experience subthemes | N | N | N |
| Altman, J. Advanced Nursing, 2021. | USA | To explore the experiences of care for pregnant and birthing people, and the **nurses** who cared for them, during the COVID-19 pandemic, with special emphasis on the impact of visitor restrictions policies. | Patients (n=15): **Nurses (n=14):** | April 2020 - August 2020 | Virtual, semi-structured interviews | Thematic analysis | 1. Policies are not equitable and disproportionately impact BIPOC families. 2. Restricting visitors has a profound impact on the experience of pregnancy and birth. 3. Recommendations for centering community in policies. | N | N | N |
| Anderson, M.  Journal of affective disorders,  2022 | USA | To understand the experiences of pregnant and postpartum women with histories of clinically elevated symptoms of depression. | Pregnant and postpartum women (n=60) | April 2020 - April 2021 | Survey questions and open-ended questions | Thematic analysis | 1) Most stressful aspects of Covid/impact on mental health; 2) Greatest worry about Covid; 3) Most useful coping strategies; 4) Positive experiences during the Covid-19 pandemic/silver linings; 5) Advice to other pregnanct and postpartum women. | N | N | N |
| Andrejek, N. *International journal of environmental research and public health,*  2021 | Canada, USA | To examine barriers and facilitators to resuming in-person psychotherapy with perinatal patients as the pandemic abates. | Perinatal participants (n=23), **physcotherapy providers (n=28), stakeholders (n-18)** | August 2020 - March 2021 | Mixed methods: Semi-structured focus groups and individual interviews | Thematic analysis | Covid-19 specific: concerns over exposure in hospital; concerns over exposure on public transit; needing to wear masks during sessions. General: Lack of childcare; transportation; planning and time constraints for new parents. | Y | N | N |
| Ashby, G. *BMC pregnancy and childbirth,* 2022 | USA | To characterize the impact of the COVID-19 pandemic on pregnant and postpartum women in a large Midwestern health system by exploring their experiences with prenatal care and support in the perinatal period. | Pregnant women (n=647) | January 1st 2020 - April 28th 2021 | COPE-IS, Single-answer, multiple-choice, and Likert Scale questions, open-answer questions. | Thematic qualitative analysis | Uncertainty; Social isolation and socialisation; Mental health; Positive reflections | Y | N | N |
| Atmuri, K. *Women and birth: journal of the Australian College of Midwives,*  2022 | Australia | To study the perspectives of pregnant women in Australia in relation to the impact of the COVID-19 pandemic on their pregnancy experience. | Pregnant women (n=15) | 1st June 2020- 19th June 2020 | Semi-structured interviews | Thematic qualitative descriptive study (Braun & Clarke) | Support for positive experience; impact on preparedness in pregnancy and beyond; facing uncertainty of a pandemic; retaining resilience and optimism. | Y | N | N |
| Badr, H. *International journal of environmental research and public health,* 2022 | Saudi Arabia | To explore the experiences of Saudi Arabian breastfeeding mothers during the COVID-19 pandemic. | Breastfeeding mothers (n=18) | March 2020 - not specified | Semi-structured, open-ended phone interviews | Thematic analysis by Polit and Beck | Breastfeeding during covid positives and negatives;support and resources; facilitators; challenges | N | N | N |
| Bayrampour, H. *CMAJ open,*  2022 | Canada | To explore the experiences of pregnant people and their responses to the COVID-19 pandemic, and to identify how health care providers can support this population. | Pregnant women (n=96). | 20 May 2020 - May 31 2020 | mixed methods:  Online survey tools: EPDS, GAD-7,  open-ended questions, Clinical diagnostic interviews via phone. | Thematic analysis | Uncertainty about birth plans and setting; added burden to existing health and social disparities; perceived or projected lack of support or limited support; concerns about early development; struggle over managing multiple demands. | N | N | N |
| Beeso, T. *Journal of patient experience,*  2021 | USA | To understand how the pandemic has altered pregnant individuals’ perceived needs and expectations about pregnancy, childbirth, and postpartum stages, based on the patient perspective and (2) to offer information to assist providers in addressing pregnant individuals’ needs for informational, structural, and social support. | Pregnant women (n=380) | April 2020- December 2020 | Online questionnaire (n=380),  Semi-structured telephone interviews (n=18) | Thematic qualitative design. | prenatal experiences; childbirth plans and expectations; priorities relating to pregnancy and childbirth during covid-19; | N | N | N |
| Breman, R. *Birth,* 2021 | USA | To describe birth experiences during the COVID- 19 pandemic cantering the birthing person's perspective | Post-partum w (n=388) | May 20th 2020 - not specified | Mixed methods. Survey and open-ended questions | Content analysis. | Institutional policies; changes in care; hospital staff interations; sub-par care; issues of support; maternal health | N | N | N |
| Cassar, E. Bachelor's dissertation, 2021 | Malta | To explore mothers’ lived experiences of antenatal education during the COVID-19 pandemic. | Postpartum women (n=9) | December 2 2020 - December 22 2020 | Virtual semi-structured interviews | Thematic qualitative analysis. | The influence of antenatal education on pregnancy, The influence of antenatal education on labour, Mothers’ preparation for the postpartum and motherhood, The overall learning experience | Y | N | N |
| Charvat, E. Journal of Family Communication, 2021 | USA | To explore how women who were pregnant during the COVID-19 pandemic (n = 21) communicatively made sense of their experience in light of their received social support. | Pregnant women, (n=21) | **Not specified** | Semi-structured interviews | Thematic qualitative analysis. | a) connecting to mitigate stress, b) drawing on others’ knowledge, c) receiving socially distant instrumental support, and d) lacking medical professional support | N | N | N |
| Choi, R. B*MC pregnancy and childbirth, 2*022 | USA, Canada, **UK,** Australia | The present study aimed to better understand the concerns of pregnant women during the beginning COVID-19 pandemic by analyzing content posted during the month of March 2020 on online pregnancy message boards hosted on WhatToExpect.com. | Pregnant women (n posts =5541) | Mar-20 | Online chat forums - whattoexpect.com | Thematic analysis | Covid-19 exposure, Labour and delivery, Social interactions, Prenatal care, Information | N | N | N |
| Claridge, A. M. *Couple and Family Psychology: Research and Practice, 2021* | USA | To examine women’s experiences of prenatal depression during the COVID-19 pandemic using a mixed-method design. | Pregnant women (n=21) | 2020 | Telephone semi-structured interview | Thematic qualitative analysis. | Fear and anxiety, mixed emotions, grief and loss | N | N | N |
| Colaceci, S. *International journal of environmental research and public health,* 2022 | Italy, Spain | To describe the impact of the pandemic on new and expectant parents in both Italy and Spain | New or expectant **Parents (n=236)** | April 2021 - June 2021 | Social media posts | Thematic qualitative analysis. | (1) care; (2) overcoming difficulties and problem-solving strategies; and (3) legislation and anti-COVID-19 measures. | N | N | N |
| Collart, C. W*omen's health reports, 2*022 | USA | To investigate the impact of these policies on pregnant patients who received outpatient prenatal care. | Pregnant women (n=40) | May 2020 - July 2020 | Semi-structured interviews | Thematic qualitative analysis. | (1) increased maternal concern, anxiety, and mental health concerns stemming from the lack of in-person partner support, (2) disappointment and lost experiences for patients seeking support from their partner during pregnancy, and (3) impact on prenatal care quality and patient experience. | Y | N | N |
| Combellick, J. *Birth, 2*022 | USA | This study aimed to identify what childbearing people needed to achieve a positive birth experience during the pandemic. | Postpartum women (n=707) | June 2020 - November 2020 | mixed methods: questionnaire, one single open-ended question | Thematic qualitative analysis. | (a) anticipatory stress and fear, (b) community birth, (c) unnecessary interventions, and (d) the pandemic care rush. | N | N | N |
| Critchlow, E. O*bstetrics and gynecology, 2*022 | USA | To describe postpartum experiences of women who gave birth during the coronavirus disease 2019 (COVID-19) pandemic, to identify short-term and long-term opportunities to address maternal–child health during this pandemic. | Women (n=30) between 3 and 10 weeks postpartum | 10 December 2020 - 1 April 2021 | Telephone interviews – photo elicitation | Content analysis | self -care, support, stressors, government resources, virtual resources, changing policies | Y | N | N |
| Davis, J. *International journal of environmental research and public health,* 2021 | Australia | To explore the relationship between emotional health and wellbeing and support needs of perinatal women during the COVID-19 pandemic, and to understand their experiences and need for support. | Pregnant or postpartum women (n=14) | November 2020 - February 2021 | Semi-structured interviews | Qualitative content analysis, thematic analysis | 1) Impact of C-19 on psychological care. 2) Isolation from friends and family. 3) Information and support needs. 4) Positive outcomes | Y | N | N |
| DeJoy, S. *Journal of midwifery & women's health, 2021* | USA | To understand childbearing persons’ decision-making during the pandemic and to illuminate their experiences giving birth in community settings | Postpartum women (n=17) | May 2020 - October 2020 | Semi-structured interviews | Thematic qualitative analysis. | Prior desire for community birth: Prior negative hospital experience Seeking the “home birth experience” Perceived susceptibility: Personal experience Natural defenses Fear of the unknown Barriers to choice: Access to maternity providers Financial difficulties Isolation: Hospital policies Telemedicine Safety bubbles | N | N | N |
| Dol J, A. Birth. 2022 | Canada | To evaluate the preliminary impact of Essential Coaching for Every Mother on maternal self-efficacy, social support, postpartum anxiety, and postpartum depression.  To explore the acceptability of the Essential Coaching for Every Mother program provided during the COVID-19 pandemic. | first time mothers (n=88) | 15 July 2020 - 19 September 2020 | Surveys - mixed methods | Thematic analysis | Support, Mood, maternal self-efficacy | N | N | N |
| Dove-Medows, E. *The Journal of perinatal & neonatal nursing,* 2022 | USA | To explore the perspectives of Black women on prenatal care, labor, and birth during the pandemic. | Pregnant black women (n=16) | May - June 2020 | Mixed methods - interviews and free text repsonses | Thematic analysis | 1) interruption of plans 2) loss of pregnancy rites of passage 3) changes to birth plans 4) fears of acquiring covid-19 | Y | N | N |
| Elling, C. *Nursing for women's health, 2*022 | USA | To examine the perceptions of **labor and delivery (L&D) nurses** and childbearing women in the postpartum period regarding a restricted visitor policy during the COVID-19 pandemic. | pregnant women (n=674) | October 2020 - March 2021 | Mixed methods. Survey and open-ended questions | Thematic analysis | More intimate, pressure is off, missing the moment of birth, needing more support, meeting siblings for the first time, kept me safe | N | N | N |
| Elliott, G. *The American journal of maternal child nursing, 2*022 | USA | To describe first-time mothers’ experiences with online social networking sites in the early postpartum period, explore how mothers use them to gain support, and to evaluate how their use can aid or hinder maternal role transition. | First time postpartum mothers (n=12) | 11 December 2020 -January 2021 | Semi-structured interviews | Thematic analysis | 1) Habits of first-time mom using social networking sites, 2) New purpose online, 3) Taking it to the moms, and 4) Impact on motherhood | N | N | N |
| Eri, T, M*idwifery,* 2022 | Norway | To explore the experiences of being pregnant, giving birth and becoming a parent in Norway during the COVID-19 pandemic. | Postpartum women (n=806) | June 2020 - July 2021 | mixed methods - open ended survey questions | Thematic analysis | 1) Pregnancy as a stressful waiting period; 2) Feeling lonely, isolated, and disempowered without their partner; 3) Sharing experiences and becoming a family; and 4) Busy postnatal care without compassion | N | N | N |
| Farrell, R. *JMIR formative research,* 2022 | USA | To support the development of successful models to serve the needs of pregnant patients, obstetric providers, and health care systems during this time. | Women receiving obstetric care at specific outpatient clinics (n=40) | May 2020 - July 2020 | Semi-structured telephone interviews | Thematic analysis | Theme 1: Perceptions of the benefits of telehealth during the pandemic. Theme 2: Reassurance that comes from in-person clinical visits with an obstetric provider. Theme 3: Added concerns about the responsibility of determining the well-being of the pregnancy at home. Theme 4: The impact of telehealth on patient experience with pregnancy and prenatal care | Y | Y | N |
| Fumagalli, S. *Women and birth : journal of the Australian College of Midwives,* 2022 | Italy | To explore childbearing experiences of COVID-19 positive mothers who gave birth in the months of March and April 2020 in a Northern Italy maternity hospital. | Covid-19 positive pregnant women (n=34) | Mid-June 2020 - End-June 2020 | Semi-structured interviews via telephone/video calls/face-to-face | Thematic analysis | Coping with unmet expectations, : reacting to the ‘new ordinary’, : ‘pandemic’ relationships, sharing a traumatic experience with long-lasting emotional impact | N | N | N |
| Geoghegan, S. *Human vaccines & immunotherapeutics,* 2021 | Ireland | To investigate the attitudes of pregnant women toward COVID-19 vaccines, so that women may be supported to make the best decision for their individual risk profiles. | pregnant women (n=300) | 4 December 2020 - 14 January 2021 | Mixed methods - survey with one open-ended question | Thematic analysis | Vaccine safety concerns and emphasis on research, the role of provider recommendation, | N | N | Y |
| Glassman, M. C*linical pediatrics, 20*22 | USA | To investigate the impact of breastfeeding support services on mothers' breastfeeding exoeriences when provided by a HCP in the paediatric medical home. | new mothers (n=28) | December 2019 - July 2020 | Mixed methods - telephone interviews | Thematic analysis | preparedness for breastfeeding, presenting issue at the MD/IBCLC visit, what about this breastfeeding consultation service contributed to mothers making an appointment, Satisfaction With the MD/IBCLC Visit and Impact of This Visit on Their Breastfeeding Experiences, Impact of MD/IBCLC Visit on Satisfaction With the Medical Home, Suggestions for Enhancing the Breastfeeding Consultation Service, Mothers’ Experiences With the Video Visit Format During the COVID-19 Pandemic, Impact of COVID-19 on Breastfeeding Experiences | Y | N | N |
| Gomez-Roas, M. *PloS one,* 2022 | USA | To identify additional challenges to healthcare interactions that emerged for low-income postpartum individuals during the pandemic | Postpartum women (n=46) | March 2020 - June 2020 | Interviews | Thematic analysis | Uncertainty about covid status, covid-19 testing, separation from newborn, visitor restrictions | Y | N | N |
| Goyal, D. *The American journal of maternal child nursing,* 2022 | USA | To identify postpartum depression risk and describe experiences of women in the fi rst 6 weeks after giving birth during the COVID-19 pandemic. | Postpartum women (n=262) | March 2020 - June 2020 | Mixed-methods - open-ended questions | Content analysis methods | Isolation and seclusion, fear anxiety and stress, grieving the loss of normal, postpartum depression, silver lining | N | N | N |
| Goyal, D. *MCN,*  2022 | USA | To explore the experiences of pregnant women who were living in the United States during the COVID-19 pandemic. | Pregnant women (n=361) | 21 May 2020 - 22 December 2020 | Survey: open-ended question | Content analysis methods | 1) losing the experience of going through pregnancy together and 2) loss of social support and expected relationship building. | N | N | N |
| Goyal, D. MCN, 2022 | USA | To explore the wellbeing, pregnancy, childbirth, and postpartum experiences of Asian American women who gave birth during the COVID-19 pandemic. | Asian-American women (n=38) who gave birth during c-19 | 12 August 2020 - 31 January 2021 | Semi-structured interview - qualitative | Thematic analysis | 1) unexpected perinatal journey, and 2) the emotional and psychological consequences of COVID-19 | N | N | N |
| Granada, S. J*ournal of immigrant and minority health,* 2022 | USA | To describe the birth experience during COVID-19 among monolingual Spanish and bilingual Spanish/English speakers. | (n=15) monolingual Spanish, (n=15) bilingual English/Spanish | September 2020 - October 2020 | Videoconference interviews | Thematic analysis | Quality of care, birth outcome, and supportive staff | N | N | N |
| Hadjigeorgiou, E. *BMC ,* 2022 | Cyprus | To examine the impact of the COVID-19 pandemic on the experiences, concerns and needs of pregnant and postpartum women in Cyprus. | pregnant and 6 month postpartum women (n=695) | July 2020 - January 2021 | Mixed-methods - survey with closed and open-ended questions | Thematic analysis | Impact on life and stress levels, perinatal care experiences, pregnant women's concerns, postpartum women's concerns, women's needs during the perinatal period | N | N | N |
| Jensen, N. *BMC pregnancy and childbirth* , 2022 | Denmark | To investigate (i) how women with recent GDM experienced COVID-19 and the first lockdown in Denmark, and (ii) the women’s risk perception and health literacy in terms of interaction with the healthcare system. | women (n=11) with recent GDM with infants aged 2-11 months old | 24 April 2020 - 26 May 2020 | Semi-structured interviews by telephone/skype | Qualitative content analysis | i) Everyday life and family well-being, ii) Worries about COVID-19 and iii) Health literacy: Health information and access to healthcare | Y | N | N |
| Keating, N. *Irish journal of medical science,* 2022 | Republic of Ireland | To explore women’s experience of pregnancy and birth in the Republic of Ireland during the COVID-19 pandemic. | Pregnant or postpartum women (n=14) | April 2020 - July 2020 | Open questions and semi-structured interviews zoom/phone | Constant comparative method | Loss of normality, Navigating the “new” maternity care system, Partners as bystanders, Balancing information, uncertainty, Unexpected benefts | N | N | N |
| Kinser, P . Midwifery , 2022 | USA | To explore the lived experience of pregnant and postpartum women in the United States during the ongoing COVID-19 pandemic. | Pregnant and postpartum women (n=54) | October 2020 - January 2020 | Semi-structured interviews via zoom | Hermeneutic phenomenological qualitative method | 1) struggles of motherhood 2) lack of healthcare resources available to address needs of pregnant and postpartum women | N | N | N |
| Kluwgant, D. *Midwifery, 2*022 | Australia | To understand the positive experiences of pregnant women during the COVID-19 pandemic. | Pregnant or postpartum women (n=20), **midwives (n=16)** | March 2020-2021 | Mixed-methods: open-ended survey questions | Content analysis methods | Valuing the mandated changes to care, Appreciating access to specific models of care, Supportive factors, Calming factors | Y | N | N |
| Kolker, S. *BMC pregnancy and childbirth,* 2021 | Canada | To explore pregnant individuals’ lived experiences as well as their psychological and behavioural responses during COVID-19 with the goal of providing useful strategies for future pandemics | Pregnant women (n=12) | June 2020 - September 2020 | Semi-structured interviews | Thematic analysis | 1) Childbearing-related challenges to everyday life; 2) Increased worry, uncertainty and fear; 3) Pervasive sense of loss; 4) Challenges accessing care; 5) Strategies for coping with pandemic stress; 6) Refections and advice to other pregnant people and health care professionals | Y | N | N |
| Linden, K. *Women and birth : journal of the Australian College of Midwives* , 2022 | Sweden | To gain a deeper understanding of how women not infected by SARS-CoV-2 experienced pregnancy during the COVID-19 pandemic in Sweden. | Pregnant women (n=14) who had not contracted covid-19. | March 2021 - April 2021 | Open-ended interviews via video/audio | Phenomenological reflective lifeworld approach | 1) dealing with uncertainties 2) being in an information echo 3) feeling socially isolated 4)facing maternity care without support 5) trusting maternal health-care services | Y | N | N |
| LoGiudice, J. *JOGNN* , 2022 | USA | To understand the experiences of women who were pregnant during the initial stage of the COVID-19 pandemic, March 2020 to May 2020, and how they coped with stress. | Pregnant women (n=185) | April 2020 - May 2020 | Mixed-methods - survey with 1 open-ended question | Content analysis methods | 1) Robbed of Enjoying the Expected Pregnancy Experiences 2) Anxiety and Fear in the Face of a Pandemic Pregnancy 3) Heightened Source of Worry With Birth on the Horizon 4) Choosing Hope. | N | N | N |
| Meaney, S. *Women and birth : journal of the Australian College of Midwives , 2022* | Republic of Ireland, USA, Ireland, **UK,** | To assess pregnant women’s satisfaction with antenatal care and social support and to examine stress-reduction strategies women used during the pandemic. | Pregnant women (n=573) | 16 June 2020 - 17 July 2020 | Mixed-methods survey - open-questions | Qualitative content analysis | 1) Altered social networks and supports due to COVID-19 2) Concerns related to Covid-19 infection 3) Juggling roles and responsibilities 4) Maternity care impacted by COVID-19 5) Occupational and financial concerns 6) Physical manifestations | Y | N | N |
| Mehl, S. *The Journal of surgical research,* 2022 | USA | To explore differences in demographics of expectant mothers evaluated pre- and post-telemedicine implementation | Pregnant women with complications by surgical fetal anomalies (n=292) | August 2021 - December 2021 | Mixed methods: semi-structured interviews | Thematic analysis | 1) convenience of telemedicine 2) resistance to telemedicine 3) technical issues of telemedicine. | Y | N | N |
| Naurin, E. E*uropean journal of public health,* 2021 | Sweden | To study how pregnant women faired **during and after** the pandemic and a focus on their health-related worries. | Pregnant women and their partners (n=6941) | 16 September 2019 - 25 August 2020 | Mixed-methods - questionnaire with open-ended questions | Thematic ananlysis | 1) social isolation 2) pregnancy and childbirth 3) work and personal economy 4) everyday life 5) worry of loved ones 6) my own health and covid-19 7) working in health care 8) society | N | N | N |
| Sunita P. Midwifery, 2021 | Republic of Ireland | To gain insight and understanding of women’s views and experiences of maternity care during the COVID-19 pandemic in Ireland. | Pregnant and postpartum women (n=19) | April-May 2020 | Semi-structured interviews via telephone | Thematic analysis | 1) navigating the system 2) at the end of the day it's just you 3)preparing for and adapting to uncertainty 4) blessing in disguise | Y | N | Y |
| Ravaldi, C. *JOACM, 2*021 | Italy | To explore the psychological impact of the COVID-19 pandemic on Italian pregnant women, especially regarding concerns and birth expectations. | Pregnant and postpartum women (n=200) | 2020 | Mixed methods - survey open-questions | MAXQDA qualitative analysis | 1) women's concerns 2) women's emotions | N | N | N |
| Redmond, M. *Journal of community psychology,* 2022 | USA | To understand COVID‐19 vaccine perceptions and decision‐making among a racially/ethnically diverse population of pregnant and lactating women in the Midwest. | Pregnant or postpartum women (n=27) | June 2020 - August 2020 | Mixed methods online survey - open-ended questions | Thematic analysis | 1) concern about vaccine safety 2) general anxiety from the pandemic 3) limited vaccine hesitancy 4) seeking knowledge | N | N | Y |
| Rice, K. C*MAJ,* 2021 | Canada | To examine how people in Canada who gave birth during the pandemic were affected by policies aimed at limiting interpersonal contact to reduce SARS-CoV-2 transmission in hospital and during the early weeks postpartum. | Pregnant and postpartum women (n=65) | June 2020 - January 2021 | Semi-structured telephone interviews | Thematic analysis | 1) negative postpartum experience in hospital 2) poor postpartum mental health 3) asking for help 4) breastfeeding problems | Y | N | N |
| Rice, K. JOACM, 2022 | Canada | To examine the impact of pandemic policy changes on experiences of pregnancy and birth; to inform understandings of medicalization,care,pregnancy, and subjectivity during times of crisis; and to critically examine the assumptions about pregnancy and birth that are sustained and produced through policy | Pregnant and postpartum women (n=67) | March 2020 - January 2021 | Semi-structured telephone interviews | Thematic analysis | 1) reduced care 2) Increased medicalisation 3) Medical intervention in response to pandemic pressures | N | Y | N |
| Rodríguez-Gallego, I. *Breastfeed,* 2022 | Spain | To explore the impact of the pandemic and of the measures adopted on breastfeeding  initiation and maintenance. | Pregnant and postpartum women (n=42) | January 2021 - May 2021 | Semi-structured telephone interviews | Thematic analysis | 1) Information received 2) Unequal support from the professionals during the pandemic 3) Social and family support on breastfeeding 4) Impact of confinement and of the social restriction measures. 5) Emotional effects of the pandemic | N | N | N |
| Ryan, A. *Midwifery,* 2022 | New Zealand | To find out the perspectives of Auckland-based mothers **and healthcare providers** on the needs and experiences of women with postnatal mental health concerns within the pandemic context | Mothers (n=8) who gave birth in the first year of the pandemic,  **healthcare professionals (n=3)** | May 2021 - July 2021 | Semi-structured interviews via video | Thematic analysis | 1) uncertainty and anxiety, 2) financial and work stress, 3) importance of the “village”, 4) inner resilience, and 5) “no one cared for mum”. | N | N | N |
| Saleh, L. T*he Journal of perinatal & neonatal nursing,* 2022 | USA | To gain insight into the experiences of women who gave birth in the United States during coronavirus disease-2019 | Pregnant and postpartum women (n = 32) | January 2021 - December 2021 | Mixed methods: online surveys & interviews via zoom | Thematic analysis | 1) expectations versus reality 2) early versus late COVID-19 experience 3) mental distress versus mental health 4) healthcare policy versus COVID-19 confusion. | N | N | N |
| Schmiedhofer, M. I*nternational journal of environmental research and public health,* 2022 | Germany | To explore the first-hand experience of the impact of the COVID-19 pandemic on mothers, **their partners, and obstetric professionals** regarding birth and obstetric care | Postpartum mothers (n=25), **partners (n=5), obstetric professionals (n=10).** | February 2021 - August 2021 | Semi-structured interviews | Qualitative content analysis | 1) How did restrictions affect phases of pregnancy? 2) Impact on roles 3) Management of burdens 4) Challenges of Covid-19 diagnosis | Y | N | N |
| Shorey, S. *Journal of clinical nursing,* 2023 | Singapore | To explore the perspectives of **parents** during the perinatal period amid the COVID-19 pandemic and explore the experiences of Singaporean parents receiving perinatal support via the Supportive Parenting App (SPA | **Parents** (n=33) | June 2021 - February 2022 | Semi-structured interviews | Thematic analysis | 1) Ups and downs of parenting 2) Perinatal care 3)What kept couples going 4) Use of technology | Y | N | N |
| Shuman, C. Maternal and child health journal, 2022 | USA | To describe the peripartum experiences of women who gave birth during the COVID-19 pandemic in the United States. | Postpartum women (n=371) | June 4 2020 - July 8 2020 | Survey - open-ended free text data | Thematic analysis | 1) Heightened emotional  distress 2) Adverse breastfeeding experiences 3) Unanticipated hospital policy changes shifted birthing plans 4) Expectation vs. reality: “mourning what the experience should have been;” 5) Surprising benefts of the COVID-19 pandemic  to the delivery and postpartum experience. | N | N | N |
| Silva-Jose, C.  *BMC,* 2022 | Spain | To explore the experiences of pregnant women who participated in an online group exercise program during the pandemic and identifies relationships with maternal mental health and well-being | Pregnant women (n=24) | March 2020 - October 2020 | Mixed-methods: Semi-structured interviews via zoom | Thematic analysis | 1) time availability 2) home confnement 3) COVID-19 | Y | N | N |
| Singla, D. *Journal of affective disorders,* 2022 | Canada, USA | To identify relevant barriers and facilitators from the perspectives of both perinatal participants and treatment providers | Perinatal women (n=23) with depressive symptoms, **treatment providers (n=28)** | March 2020 - January 2021 | Semi-structured interviews online, focus groups and case studies | Thematic analysis | 1) Participants receiving behavioural analysis during covid-19 2) Providers delivering behavioural analysis during covid-19 | N | N | N |
| Siwik, E. *Journal of human lactation, 2*022 | Canada | To explore the experiences of at-risk postpartum breastfeeding women in accessing formal and informal  breastfeeding social support during the COVID-19 pandemic | Postpartum women (n=7) from an at-risk population attending a postpartum clinic | March 10 2020 - September 1 2020 | Mixed methods: online survey and a semi-structured interview via telephone | Interpretive description approach | 1) Barriers as a result of the Covid-19 pandemic 2) Facilitators to breastfeeding as a result of the pandemic | Y | N | N |
| Spach, N. *Women's health reports,* 2022 | USA | To characterize the emotional effects of the COVID-19 pandemic on pregnant and recently pregnant patients who had either suspected or confirmed COVID-19 infection during the initial 6 months of the pandemic. | Pregnant/ recently postpartum women (n=20) who has/ suspected Covid-19 infection. | March 2020 - August 2020 | Semi-structured telephone interviews | Thematic analysis | 1) Risk 2) Protection 3) Change | N | N | N |
| Spatz, D. *MCN.* 2021 | USA | To teach  health care providers maternal perceptions and to proactively work to ensure we provide sound  anticipatory guidance, enhance our communication, and improve provision of evidence-based lactation care and  support | First-time mothers (n=3) who were breastfeeding | Feb-21 | Case study interviews | Thematic analysis | 1) Recommendations changing every day, 2) Guilt, concern, and stress, 3) In-person versus telehealth visits, 4) Missing time with family and friends, and 5) Silver linings | N | N | N |
| Stirling C. *Reprod Health,* 2021 | Canada | To understand Syrian refugee women’s experiences accessing postnatal healthcare services and supports during the COVID-19 pandemic | Postnatal women (n=8) | Mar-20 | Semi-structured virtual interviews | Constructivist grounded theory | 1) the impacts of COVID-19 on postnatal healthcare 2) loss of informal support 3) grief and anxiety. | Y | N | N |
| Sullivan, E. J*ournal of midwifery & women's health,* 2022 | Canada | To explore rural residents' perinatal experiences during the initial months of Covid-19 | Perinatal women (n=16) | Mar-20 | Semi-structured interviews and focus groups | Thematic analysis | 1) Perceived risk of infection 2) navigating uncertainty 3) experience of care received 4) resilience and silver linings. | Y | N | N |
| Sweet, L. *Midwifery,* 2021 | Australia | To describe childbearing women’s experiences of becoming a mother during the COVID-19 pandemic in Australia | New mothers (n=27) | March 2020 - June 2020 | Semi-structured telephone/zoom interviews | Thematic analysis | 1) ‘going it alone – having a baby was an isolating experience’ 2) ‘advocating for self or others’ 3) ‘finding a way through’ 4) ‘keeping safe’. | N | N | N |
| Sweet, Li. JOACM, 2022 | Austrailia | To explore and describe childbearing women’s experiences of receiving maternity care during the COVID-19  pandemic in Australia. | Perinatal women (n=27) | Feb-21 | Semi-structured online interviews | Thematic analysis | 1) navigating a changing health system 2) desiring choice and control 3) experiencing infection prevention measures | Y | Y | N |
| Tavares, I. *Frontiers in psychology,* 2021 | Portugal | To investigate individual and relational well-being of women and men who were expecting their first child during the first months of the COVID-19 pandemic in Portugal | **women n=198, men n=118** in a committed relationship who were expecting or 6 moths postpartum | March 2020 - November 2020 | Mixed methods: one open ended question | Thematic analysis | 1) Individual Changes 2) Relational Changes | N | N | N |
| Testoni, I. *Frontiers in psychology,*  2022 | Italy | To research the psychological impact of the COVID-19 pandemic on couples grieving for perinatal loss, this research aimed to survey this experience. | 21 parents **(women n = 16, men n = 5)** who experienced a perinatal loss | March 2020 - March 2021 | Mixed methods: semi-structured online interviews | Thematic analysis | 1) Psychological Complexity of Bereavement 2) The Impact of COVID-19 3) Disenfranchisement vs. Support | N | N | N |
| Vance, A. *BMC pediatrics* , 2021 | USA | To describe the lived experience of parents who had an infant in the NICU in the context of the COVID-19 pandemic to inform healthcare providers and policy makers for future development of policies and care planning. | **Parents (n=169)** who had an infant in NICU | May 2020 - July 2020 | Survey: online open-ended questions | Thematic analysis | 1) parents’ NICU experiences during the COVID-19 pandemic were emotionally isolating and overwhelming 2) policy changes restricting parental presence created disruptions to the family unit and limited family-centered care 3) interactions with NICU providers intensified or alleviated emotional distress felt by parents. | N | N | N |
| Vermeulen, JSAM, 2022 | Belgium | To explore how pregnant women and women  who have recently given birth experienced the pandemic period. | Perinatal women (n=556) | April 22 2020 - June 15 2020 | Survey: onlineopen-ended question | Thematic analysis | 1) fear of contamination 2) feeling isolated and unsupported 3) not able to share  experiences 4) disrupted care 5) feeling unprepared 6) experience a peaceful period | Y | N | N |
| Wilson, A. *Birth,* 2022 | Australia | This study aimed  to explore women's experiences of receiving maternity care during the COVID-19  pandemic in Australia. | Perinatal women (n=3364) | Mar-20 | Mixed methods online Survey: open-ended questions | Content analysis | 1) Deprived of anticipated maternity experiences 2) Feelings of distress 3) Doing it alone | N | N | N |
| Yip, K. *IJERPH,* 2022, | Hong Kong | To explore the experiences of women without Covid-19 in breastfeeding during the pandemic | Breastfeeding mothers (n=20) | December 2021 - February 2022 | Semi-structured interviews | Colaizzi's phenomenological methodology | 1) positive influences on breastfeeding support during COVID-19 2) negative influences on breastfeeding support during COVID-19. | N | N | N |
| Young, A. *BMC,* 2022 | New Zealand | To explore what pregnant/recently pregnant Māori and Pacifc women knew about immunisation during pregnancy and what factors infuenced their decision to be vaccinated. | Pregnant women (n=15) | May 2021 - August 2021 | Semi-structured interviews | Thematic analysis | 1) Gaps in  awareness 2) Vaccination: Possible protection vs. potential  harms 3) Infection: Perceived potential harm and risk  of infection | N | N | Y |
| **Updated search (Oct 2022 – June 2024)** | | | | | | | | | | |
| Atchan, M. WOMBI, 2023. | Australia, New Zealand | To explore the early parenting and infant feeding experiences of new mothers from Australia and Aotearoa New Zealand in the context of a pandemic. | 27 mothers who gave birth during the first wave of the COVID-19 pandemic | September - October 2020 | Semi-structured interviews | Thematic analysis | 1) Feeding decisions and practices 2) The COVID-19 breastfeeding experience 3) Experiences of support 4) Psychological impact | Y | N | N |
| Church, A. Perspect. Sex. Reprod. Health, 2023 | USA | To examine women's perceptions and experiences of solitary support, particularly from male partners, during labor and delivery. | 12 women who gave birth after the onset of the COVID-19 pandemic and all opted to have their male partner as their solitary support person | April 2020 - August 2021 | Semi-structured interviews | Thematic analysis | 1) Improved relationships and communication 2) Privacy versus isolation 3) Fear and anxiety 4) | N | N | N |
| Dixon, L. et al. J NZ Coll Midwives, 2023 | New Zealand | To explore the experiences of women who were pregnant, giving birth and/or managing the early weeks of motherhood during the 2020 COVID-19 alert levels 3 and 4 in Aotearoa New Zealand. | 17 women | Not stated | Semi-structured interviews | Thematic analysis | 1) Relationship with my midwife 2) Disruption to care 3) Isolation 4) Undisturbed space | Y | N | N |
| Dol, J. J. Nurs. Scholarsh, 2022. | Canada | To (1) compare changes in parenting self-efficacy, social support, postpartum anxiety, and postpartum depression in Canadian women before and during the early COVID-19 pandemic; (2) explore how women with a newborn felt during the pandemic; (3) explore ways that women coped with challenges faced | 561 women completed the survey, and 331 women during the pandemic. At time point 1Who an infant 6 months old or less in one of the three Eastern Canadian Maritime provinces | Prior to the pandemic (October 1, 2019–January 1, 2020), a survey was conducted with women living in the Maritime provinces (Cohort 1). Following the first wave of COVID-19 in Canada, a similar survey was conducted between August 1 and October 31, 2020 (Cohort 2). | Online Mixed-methods survey | Thematic analysis | Women mentioned several difficulties they experienced as a result of COVID-19 restrictions, including lack of support from family and friends, fear of COVID-19 exposure, feeling isolated and uncertain, negative impact on perinatal care experience, and hospital restrictions. Women also reflected on what helped them cope during this challenging period and acknowledged that this came from having support from partners, support from families, in-person/virtual support, as well as engaging in self-care and the low epidemiology of COVID-19 during the summer of 2020. | Y | N | N |
| Jaffe, E. WHI, 2022 | USA | To characterize the range of ways that the early months of the COVID-19 pandemic affected pregnancy, childbirth, and postpartum care experiences. | Pregnant and recently pregnant patients (n = 20) from obstetrics and gynecology clinical sites associated with Massachusetts General Hospital. | April 2020- August 2020 | Semi-structured interviews | Not clearly stated- Coded for emergent themes | Three themes were identified across narratives of pregnancy, birth, and postpartum care: patient perceptions of diminished access to care, stigma due to COVID-19 infection, and limited capacity of providers to honor patient preferences. | Y | N | N |
| Rice, M. Matern Child Health J, 2024. | USA | To better understand the experiences of Black pregnant women during COVID-19, we examined Black pregnant clients’ and doulas’ experiences with perinatal support services amid COVID-19’s social distancing protocols. | 29 Black women who were pregnant or gave birth during the pandemic + Doulas who provided care | fall of 2020 and winter of 2021 | Semi-structured interviews | Not clearly stated- Coded for emergent themes | Three key themes were identified: (1) Clients experienced increased social isolation; (2) Doulas’ exclusion from medical visits limited women’s access to support and advocacy; (3) Doula support as a sisterhood helped clients mitigate effects of COVID isolation. | N | N | N |
| Rokicki, S. Matern Child Health J, 2023. | USA | To explore the lived experiences of women with perinatal depression and anxiety to elucidate their perceptions of how the pandemic influenced their mental health and access to care | 14 women with self-reported perinatal depression or anxiety who were pregnant or within one year postpartum | March-October 2021 | Semi-structured interviews | Thematic analysis | 1) *Negative impacts of COVID-19 on symptoms of depression and anxiety*  2) *Negative impacts of COVID-19 on access to and quality of health care*  3) *Positive impacts of COVID-19 on mental health* | Y | N | N |

### **Table S4**: Critical appraisal of studies

| **Ref** | **A. Aims and objectives clearly reported** | **B. Adequately described the context of research (in reference to methods, not background)** | **C. Adequately described the sample and sampling methods** | **D. Adequately described the data collection methods** | **E. Adequately described the data analysis methods** | **F. Reliability of the data collection tools** | **G. Validity of the data collection tools** | **H. Reliability of the data analysis** | **I. Validity of the data analysis** | **J. Used the appropriate data collection methods to allow for expression of views** | **K. Used the appropriate methods for ensuring the analysis was grounded in the views** | **L. Actively involved the participants in the design and conduct of the study.** |
| --- | --- | --- | --- | --- | --- | --- | --- | --- | --- | --- | --- | --- |
| Ajayi, MCN, 2021 | Y | Y | Y | Y | Y | Y | Y | Y | Y | P | P | N |
| Altman, J. Advanced Nursing, 2021 | Y | P | Y | Y | Y | Y | Y | Y | Y | Y | Y | N |
| Anderson.  Journal of affective disorders,  2022 | Y | Y | Y | Y | Y | Y | Y | Y | Y | Y | Y | N |
| Andrejek, N. *International journal of environmental research and public health,*2021 | Y | Y | Y | Y | Y | Y | Y | Y | Y | Y | Y | N |
| Ashby, G. *BMC pregnancy and childbirth,* 2022 | P | Y | P | N | Y | P | P | Y | Y | P | Y | N |
| Atmuri, K. *Women and birth: journal of the Australian College of Midwives,*  2022 | Y | Y | Y | Y | Y | Y | Y | Y | Y | Y | Y | N |
| Badr, H. *International journal of environmental research and public health,* 2022 | Y | Y | Y | Y | Y | Y | Y | Y | Y | Y | Y | Y |
| Bayrampour, H. *CMAJ open,* 2022 | Y | Y | P | P | Y | Y | Y | Y | Y | Y | Y | N |
| Beeso, T. *Journal of patient experience,* 2021 | Y | Y | Y | P | P | Y | Y | P | P | P | Y | N |
| Breman, R. *Birth,* 2021 | Y | Y | Y | Y | Y | Y | Y | Y | Y | Y | Y | N |
| Cassar, E. Bachelor's dissertation, 2021 | Y | Y | Y | N | N | P | P | P | P | Y | P | N |
| Charvat, E. Journal of Family Communication, 2021 | Y | P | P | Y | Y | Y | Y | Y | Y | Y | Y | Y |
| Choi, R. B*MC pregnancy and childbirth, 2*022 | Y | P | N | Y | Y | Y | Y | Y | Y | P | P | N |
| Claridge, A. M. *Couple and Family Psychology: Research and Practice, 2021* | Y | Y | Y | P | P | P | P | P | P | Y | Y | N |
| Colaceci, S. *International journal of environmental research and public health,* 2022 | Y | P | N | Y | Y | Y | Y | Y | Y | N | P | N |
| Collart, C. W*omen's health reports, 2*022 | Y | Y | Y | P | P | Y | Y | Y | Y | Y | Y | N |
| Combellick, J. *Birth, 2*022 | Y | Y | Y | Y | Y | Y | Y | Y | Y | P | Y | N |
| Critchlow, E. O*bstetrics and gynecology, 2*022 | Y | Y | Y | Y | Y | Y | Y | Y | Y | Y | Y | N |
| Davis, J. *International journal of environmental research and public health,* 2021 | Y | Y | Y | Y | Y | Y | Y | Y | Y | Y | Y | Y |
| DeJoy, S. *Journal of midwifery & women's health, 2021* | Y | Y | Y | N | Y | P | P | Y | Y | Y | Y | N |
| Dol J, A. Birth. 2022 | Y | Y | P | P | N | N | P | N | N | P | P | Y |
| Dove-Medows, E. *The Journal of perinatal & neonatal nursing,* 2022 | Y | Y | Y | Y | Y | Y | Y | Y | Y | Y | Y | N |
| Elling, C. *Nursing for women's health, 2*022 | Y | Y | Y | Y | N | Y | Y | P | P | Y | P | N |
| Elliott, G. *The American journal of maternal child nursing, 2*022 | Y | Y | Y | Y | Y | Y | Y | Y | Y | Y | Y | N |
| Eri, T, M*idwifery,* 2022 | Y | Y | Y | Y | Y | Y | Y | Y | Y | Y | Y | N |
| Farrell, R. *JMIR formative research,* 2022 | Y | Y | Y | P | Y | P | P | Y | Y | Y | Y | Y |
| Fumagalli, S. *Women and birth : journal of the Australian College of Midwives,* 2022 | Y | Y | Y | P | P | P | P | P | P | Y | Y | N |
| Geoghegan, S. *Human vaccines & immunotherapeutics,* 2021 | P | Y | Y | Y | Y | Y | Y | Y | Y | Y | Y | N |
| Glassman, M. C*linical pediatrics, 20*22 | Y | Y | P | Y | P | Y | Y | P | P | Y | P | N |
| Gomez-Roas, M. *PloS one,* 2022 | Y | Y | Y | Y | Y | Y | Y | Y | Y | Y | Y | N |
| Goyal, D. *The American journal of maternal child nursing,* 2022 | Y | Y | Y | Y | P | Y | Y | P | P | Y | Y | N |
| Goyal, D. *MCN,*  2022 | Y | Y | P | Y | P | Y | Y | P | P | Y | Y | N |
| Goyal, D. MCN, 2022 | Y | Y | Y | Y | P | Y | Y | P | P | Y | Y | N |
| Granada, S. J*ournal of immigrant and minority health,* 2022 | P | Y | Y | P | P | P | P | P | P | Y | Y | N |
| Hadjigeorgiou, E. *BMC ,* 2022 | Y | Y | Y | Y | P | Y | Y | P | P | Y | Y | N |
| Jensen, N. *BMC pregnancy and childbirth* , 2022 | Y | Y | Y | Y | Y | Y | Y | Y | Y | Y | Y | N |
| Keating, N. *Irish journal of medical science,* 2022 | Y | Y | Y | P | Y | P | P | Y | Y | Y | Y | N |
| Kinser, P . Midwifery , 2022 | Y | Y | Y | Y | Y | Y | Y | Y | Y | Y | Y | N |
| Kluwgant, D. *Midwifery, 2*022 | Y | Y | Y | Y | Y | Y | Y | Y | Y | Y | Y | N |
| Kolker, S. *BMC pregnancy and childbirth,* 2021 | Y | Y | Y | P | Y | Y | Y | Y | Y | Y | Y | N |
| Linden, K. *Women and birth : journal of the Australian College of Midwives* , 2022 | Y | Y | Y | Y | Y | Y | Y | Y | Y | Y | Y | N |
| LoGiudice, J. *JOGNN* , 2022 | Y | Y | Y | Y | N | Y | Y | N | P | Y | P | N |
| Meaney, S. *Women and birth : journal of the Australian College of Midwives , 2022* | Y | Y | Y | Y | Y | Y | Y | Y | Y | Y | Y | N |
| Mehl, S. *The Journal of surgical research,* 2022 | Y | Y | P | P | P | P | Y | P | Y | Y | Y | N |
| Naurin, E. E*uropean journal of public health,* 2021 | Y | Y | P | Y | N | Y | Y | N | N | Y | P | N |
| Sunita P. Midwifery, 2021 | Y | Y | Y | Y | Y | Y | Y | Y | Y | Y | Y | Y |
| Ravaldi, C. *JOACM, 2*021 | Y | Y | Y | N | N | N | N | N | N | N | N | N |
| Redmond, M. *Journal of community psychology,* 2022 | Y | Y | Y | P | P | P | P | P | P | P | P | N |
| Rice, K. C*MAJ,* 2021 | Y | Y | Y | P | Y | P | P | Y | Y | Y | Y | N |
| Rice, K. JOACM, 2022 | Y | Y | Y | P | Y | P | P | Y | Y | P | Y | N |
| Rodríguez-Gallego, I. *Breastfeed,* 2022 | Y | Y | Y | Y | Y | Y | Y | Y | Y | Y | Y | Y |
| Ryan, A. *Midwifery,* 2022 | Y | Y | Y | Y | Y | Y | Y | Y | Y | Y | Y | Y |
| Saleh, L. T*he Journal of perinatal & neonatal nursing,* 2022 | Y | Y | Y | P | Y | P | P | Y | Y | Y | Y | N |
| Schmiedhofer, M. I*nternational journal of environmental research and public health,* 2022 | Y | Y | P | Y | Y | Y | Y | Y | Y | Y | Y | Y |
| Shorey, S. *Journal of clinical nursing,* 2023 | Y | Y | Y | P | P | P | Y | P | Y | Y | Y | N |
| Shuman, C. Maternal and child health journal, 2022 | Y | Y | Y | Y | P | Y | Y | P | P | Y | Y | N |
| Silva-Jose, C.  *BMC,* 2022 | Y | Y | Y | P | Y | Y | Y | Y | Y | Y | Y | Y |
| Singla, D. *Journal of affective disorders,* 2022 | Y | Y | Y | P | Y | Y | Y | Y | Y | Y | Y | N |
| Siwik, E. *Journal of human lactation, 2*022 | Y | Y | P | P | Y | P | P | Y | Y | Y | Y | N |
| Spach, N. *Women's health reports,* 2022 | Y | Y | Y | Y | P | Y | Y | P | P | Y | Y | N |
| Spatz, D. *MCN.* 2021 | P | P | Y | N | N | P | P | N | N | P | P | Y |
| Stirling C. *Reprod Health,* 2021 | Y | Y | Y | Y | Y | Y | Y | Y | Y | Y | Y | N |
| Sullivan, E. J*ournal of midwifery & women's health,* 2022 | Y | Y | Y | P | P | P | P | P | P | Y | Y | N |
| Sweet, L. *Midwifery,* 2021 | Y | Y | P | Y | P | P | Y | P | P | Y | Y | N |
| Sweet, L. *Midwifery,* 2021 | Y | Y | P | Y | P | P | Y | P | P | Y | Y | N |
| Tavares, I. *Frontiers in psychology,* 2021 | P | Y | Y | Y | Y | Y | Y | Y | Y | Y | Y | N |
| Testoni, I. *Frontiers in psychology,*  2022 | Y | Y | Y | P | Y | P | P | Y | Y | Y | Y | N |
| v | Y | Y | Y | Y | P | Y | Y | P | P | Y | Y | N |
| Vermeulen, JSAM, 2022 | Y | Y | P | Y | Y | Y | Y | Y | Y | Y | Y | N |
| Wilson, A. *Birth,* 2022 | Y | Y | Y | Y | P | Y | Y | P | P | Y | Y | N |
| Yip, K. *IJERPH,* 2022, | Y | Y | Y | Y | Y | Y | Y | Y | Y | Y | Y | Y |
| Young, A. *BMC,* 2022 | Y | Y | P | P | Y | Y | Y | Y | Y | Y | Y | Y |
| **Updated search (Oct 2022 – June 2024)** | | | | | | | | | | | | |
| Atchan, M. WOMBI, 2023. | Y | Y | Y | Y | Y | Y | Y | Y | Y | Y | Y | N |
| Church, A. Perspect. Sex. Reprod. Health, 2023 | Y | Y | Y | Y | Y | Y | Y | Y | Y | Y | Y | N |
| Dixon, L. et al. J NZ Coll Midwives, 2023 | Y | Y | Y | Y | Y | Y | Y | Y | Y | Y | Y | N |
| Dol, J. J. Nurs. Scholarsh, 2022. | Y | Y | Y | P | P | P | P | N | N | P | P | N |
| Jaffe, E. WHI, 2022 | Y | Y | Y | Y | N | Y | Y | N | N | Y | P | N |
| Rice, M. Matern Child Health J, 2024. | Y | Y | Y | Y | N | Y | Y | N | N | Y | P | P |
| Rokicki, S. Matern Child Health J, 2023. | Y | Y | Y | Y | Y | Y | Y | Y | Y | Y | Y | N |
